# Development and Evaluation of a Functional Food Consumption Index (FunFoCI) in Adults

**DOI:** 10.3390/nu18060895

**Published:** 2026-03-12

**Authors:** Gülden Arman, Aslı Akyol

**Affiliations:** 1 Department of Nutrition and Dietetics, Faculty of Health Sciences, Artvin Çoruh University, Artvin 08000, Turkey; 2 Department of Nutrition and Dietetics, Faculty of Health Sciences, Hacettepe University, Ankara 06100, Turkey; asli.akyol@hacettepe.edu.tr

**Keywords:** functional foods, functional food consumption index (FunFoCI), diet quality index-international (DQI-I), healthy eating index-2015 (HEI-2015), food frequency questionnaire (FFQ)

## Abstract

Background/Objectives: Functional foods are widely discussed in nutrition research, yet their consumption is rarely quantified using a standardized, food-based metric. We developed the Functional Food Consumption Index (FunFoCI) and conducted an initial evaluation of its performance in adults. Methods: In this cross-sectional study, 500 adults (≥18 years, 286 women, 214 men) were assessed using a 210-item quantitative food frequency questionnaire (FFQ) and a 3 day food record (FR). Candidate index foods were evaluated by five experts, using a 4-point Likert scale to establish content validity, and the finalized FunFoCI comprised 100 foods across nine groups: fruits; vegetables; whole grains; legumes; nuts and oilseeds; fermented foods and products; animal-based foods; functional oils; and spices, herbal teas, and functional beverages. FunFoCI scoring used a sample distribution-based percentile approach, including modifications for zero-inflated or sparsely consumed items, followed by group-level normalization (0–1), equal weighting across nine groups, and rescaling to 0–100. FR data were used to examine the between-method feasibility of the scoring approach. The convergent validity was assessed via correlation analyses, with the Diet Quality Index-International (DQI-I) and Healthy Eating Index-2015 (HEI-2015) derived from both FFQ and FR data, and additional correlation analyses and robustness checks were conducted to examine associations among key study variables. Known group patterns were examined across sociodemographic, lifestyle, and anthropometric characteristics. Results: Content evaluation supported index coverage (S-CVI/Ave = 0.912; S-CVI/UA = 0.877; mean modified kappa = 0.899). The mean FunFoCI total scores were 32.68 ± 11.92 (FFQ) and 13.29 ± 4.65 (FR). Participants were classified into low (32.8%, *n* = 164), moderate (33.0%, *n* = 165), and high (34.2%, *n* = 171) FunFoCI categories. FunFoCI correlated with FFQ-derived DQI-I and HEI-2015 (r = 0.367 and r = 0.368; both *p* < 0.001), and both indices increased across ascending FunFoCI total scores (*p* < 0.001). The FFQ-derived FunFoCI total score was correlated with the FR-derived FunFoCI score (r = 0.294; *p* < 0.001). FunFoCI scores showed differences across participant sociodemographic, lifestyle and anthropometric characteristics. Conclusions: FunFoCI is a newly developed, expert-reviewed, food-based index with transparent, sample distribution-based scoring and normalized aggregation. Its initial evaluation supports its use for the standardized quantification of relative functional food consumption in adults, while further studies should assess the reliability and external validation criteria in other populations and study designs.

## 1. Introduction

Health is a multidimensional state encompassing optimal physical, psychological and cognitive well-being [1]. As living standards improve, health perceptions and dietary behaviors continue to evolve, and greater attention has been directed toward healthy lifestyles and adequate nutrition, which are closely associated with increased life expectancy [2]. Nutrition is not only about meeting nutrient and energy requirements, but also about maximizing the health benefits derived from foods. In parallel, interest in healthy living and demand for functional foods have increased. Consistent with this trend, and depending on the market definition and product scope, market projections estimate that the global functional foods market may approach USD 586.1 billion by 2030 [3].

The concept of functional foods emerged in Japan in the 1980s [4]. However, the use of foods for therapeutic purposes has a long history in ancient cultures, including China [5]. Although definitions differ across countries and institutions, functional foods are generally described as foods and beverages, whether natural, processed or fortified, that are consumed as part of a habitual diet and provide health benefits beyond basic nutrition [1,6,7,8,9]. Evidence suggests that these benefits are largely mediated by bioactive components that are naturally present in foods, including polyphenols, peptides and other compounds with potential antioxidant, anti-inflammatory, antimicrobial, and metabolism-modulating properties [10,11,12,13,14,15,16]. For instance, grape bioactives such as resveratrol and anthocyanins have been associated with protective effects against oxidative stress and inflammation, particularly in relation to cardiovascular disease [10]. Similarly, milk proteins and their derived peptides have been identified as bioactive compounds [16]. Consequently, much of the existing literature has focused on isolated bioactive compounds and extract-based or fortified formulations, with a particular focus on bioactivity, bioavailability, and safety or toxicity considerations, rather than dietary functional food intake assessed from daily consumption [17,18,19,20,21,22]. Functional foods commonly include plant foods that are rich in bioactives, such as fruits, vegetables, whole grains, legumes, nuts, oilseeds and oils, as well as animal sources such as fish and seafood, and foods containing microorganisms, including probiotics [23,24,25]. Although reference intakes and/or safety thresholds have been proposed for certain bioactive components, like green tea catechins, there is currently no consensus on food-based reference intake values or cut-off points that classify the proportion of functional foods within the daily diet [22].

The lack of a standard definition also creates uncertainty regarding appropriate intake levels for functional foods [26], making it difficult to estimate daily intake within habitual diets [8]. Most research on functional food consumption has focused on consumer perceptions, awareness, attitudes, preferences or market trends [27,28,29]. However, no standardized tool has been identified that quantifies functional food consumption both as an intake amount and as a composite score reflecting the contribution of functional foods to the habitual diet. Therefore, a standardized method is needed to assess functional food consumption at both individual and group levels. This study aims to develop a food-based, comprehensive index for quantifying and classifying patterns of functional food consumption in the study sample using dietary intake data, based on the reported amounts of foods consumed. Such an index would enable standardized comparisons across studies and support research examining links between functional foods intake and health outcomes.

## 2. Materials and Methods

### 2.1. Study Design and Participants

This cross-sectional study aimed to develop a Functional Food Consumption Index (FunFoCI) and to examine convergent validity, using established diet quality indices. The study was conducted in Turkey from September 2022 to September 2025. However, participant recruitment and dietary data collection were completed within a single 12-month window. Data were collected by trained dietitians using structured interviews, conducted in person or remotely (real-time video). All data were interviewer-entered; no self-completed online forms were used. The interviews were administered by Department of Nutrition and Dietetics at Hacettepe University (Ankara, Turkey) and Artvin Çoruh University (Artvin, Turkey). Ethics approval was obtained from the Hacettepe University Non-Interventional Clinical Research Ethics Committee (registration No.: GO21/1204).

Adults aged ≥18 years were eligible. A total of 500 volunteers participated, including 286 female and 214 male participants. Written informed consent was obtained from all participants prior to data collection. Exclusion criteria included being bedridden; pregnancy or lactation; adherence to a vegetarian diet; and medical conditions requiring dietary modification, as self-reported by participants, including celiac disease, gluten intolerance, lactose intolerance, diabetes mellitus, or chronic kidney disease. Participants were excluded if they reported restricting specific foods for any reason, followed restrictive dietary programs (for example, a weight-loss diet), or withdrew before completing the study procedures.

### 2.2. Data Collection and Measurements

#### 2.2.1. Anthropometrics

Body weight, height, waist circumference, and hip circumference were measured using standardized procedures [30]. Body weight was measured using a digital scale with 0.1 kg sensitivity (Arzum AR553 Fitsense, Arzum, Istanbul, Turkey) [30]. Body mass index (BMI), waist to hip ratio (WHR), and waist to height ratio (WHtR) were calculated. BMI was classified according to World Health Organization (WHO) criteria [31,32], and waist circumference and WHR were classified using sex-specific WHO cut-offs [31,32]. WHtR was evaluated using the cutoffs proposed by Ashwell et al., where 0.40–0.50 indicates the normal range and values > 0.60 indicate elevated chronic disease risk [33,34].

#### 2.2.2. Dietary Assessment

Dietary intake was assessed using a quantitative food frequency questionnaire (FFQ) and a three-day food record (FR). The FFQ assessed usual dietary intake over the previous year, accounting for seasonal consumption. It comprised 210 food items across 14 sections. Frequency categories were standardized as follows: at every meal, daily, 5–6 days per week, 3–4 days per week, 1–2 days per week, once per 15 days, monthly, and never. The FR comprised three non-consecutive days (two weekdays and one weekend day), and the mean daily intake was calculated. Only participants with complete FFQ data for all index items and complete 3 day food records were included. Zero intake values represented non-consumption, rather than missing data. Dietary data were collected by a dietitian using the Turkish Food Photograph Catalog to determine the actual quantities of food consumption by respondents [35]. The quantities of foods consumed away from home were estimated using Standard Recipes [36], and packaged foods were quantified using label information. Dietary data obtained from the FFQ and FR were analyzed using BeBiS software (Nutrition Information System version 9.1; Dr. J. Erhardt, Stuttgart, Germany) to calculate food amounts, energy, and macronutrient and micronutrient intakes [37].

### 2.3. Development of the Functional Food Consumption Index (FunFoCI)

#### 2.3.1. Conceptual Framework and Functional Food Selection

The primary aim of FunFoCI was instrument development to characterize consumption patterns, rather than to establish causal health effects. In this study, functional foods were defined as foods or beverages that were consumed as part of the habitual diet (excluding supplements and pharmaceutical preparations) that are supported by at least one line of human-relevant evidence in the literature or by a recognized authoritative evaluation indicating a plausible health-related function beyond basic nutrition. Candidate items were shortlisted based on: (i) evidence suggesting a potential health benefit, (ii) feasibility of grouping by similar intake patterns or expected mechanisms, (iii) accessibility and habitual consumption, (iv) relevance to the Turkish diet with cross-cultural availability, and (v) a preference for natural or minimally processed forms (such as fermented foods), rather than supplements or commercial products with variable formulations.

The stepwise selection of functional foods was performed as follows:

Firstly, a literature search was conducted in Web of Science, PubMed, and Scopus without year restrictions, using Turkish and English terms including: (“healthy food” OR “ healthful food*” OR “healthy eating” OR “healthy diet”) AND (consum* OR intake* OR “dietary intake”), (“functional foods” OR food OR beverage OR nutrition OR diet) AND (“health effects “OR “health outcomes”), (“functional foods” OR “fortified food”) AND (index OR indices OR score OR assessment OR instrument OR tool), (“functional foods” OR “fortified food”) AND (“health claims” OR “nutrition claims”)”.

Secondly, a candidate item was eligible for inclusion if at least one of the following criteria was met:

1. Evidence from a systematic review or meta-analysis reporting beneficial effects or associations on relevant health outcomes;
2. Evidence from a randomized controlled trial indicating beneficial effects in humans;
3. A published evaluation or scientific opinion from a recognized authority (e.g., EFSA) indicating a plausible health-related function or substantiated claim;
4. At least one human study (interventional or observational) reporting a beneficial association/effect on a relevant health outcome or biomarker.

Items supported only by in vitro or animal evidence, or solely by marketing claims without human-relevant support, were not eligible. To ensure consistent dietary assessment and to avoid brand-dependent misclassification, eligible items were restricted to natural or minimally processed forms with stable composition.

Beverages were only included if they could be consistently defined at the category level and met the minimum evidence requirements above for example, unsweetened tea/coffee, and, where relevant, fermented dairy beverages such as plain kefir. Sugar-sweetened beverages, energy drinks, and products that are primarily defined by marketing claims or highly variable formulations were excluded. When both sweetened and unsweetened forms existed, only the unsweetened forms were eligible. Beverages were quantified in milliliters (mL), and tea, coffee, and herbal tea were evaluated based on their consumed volume (mL).

To prevent double counting, the intake of a given food was aggregated across comparable edible forms with stable composition (e.g., raw, cooked, dried, pureed, frozen forms of the same food). Furthermore, foods that could plausibly fit more than one prespecified food group were assigned to a single group to avoid double counting, based on predominant dietary use or consumption patterns in the population and the study’s conceptual framework. For example, yogurt, which could be classified as both an animal-derived and fermented food, was grouped under fermented foods. Mixed dishes and packaged products were decomposed into ingredient-level components based on standard recipes and/or label information, and component amounts were allocated to corresponding single-food item(s). For example, a 250 g serving of lentil soup was decomposed into lentils, onion, wheat flour, and sunflower oil, according to a standard recipe; only the eligible components (e.g., lentils, onion) contributed to their respective food groups, whereas excluded components (e.g., refined wheat flour) were not scored. Highly heterogeneous commercially formulated or packed products were excluded unless they could be clearly defined and disaggregated at the ingredient level.

Candidate items meeting the above rules were then evaluated by experts, using a predefined content validity approach.

#### 2.3.2. Food Items Content Validity

The content validity of each functional food item candidate was assessed through expert evaluation. An initial pool of 114 food items was rated independently by five experts from Nutrition and Dietetics and Public Health, using a 4-point Likert scale (1 = not representative, 2 = representative with major revision, 3 = representative, 4 = highly representative). The experts comprised five doctoral-level academics with at least five years of professional experience in their respective fields, including four experts in Nutrition and Dietetics and one expert in Public Health. Among them, one Nutrition and Dietetics expert and one Public Health expert had prior experience in scale development. Experts completed ratings independently and did not have access to each other’s ratings. Experts also provided qualitative comments to support item refinement. Prior to calculating the content validity index (CVI), ratings of 3 or 4 were prespecified as indicating relevance/representativeness. The item-level content validity index (I-CVI), the scale-level content validity index using the average method (S-CVI/Ave), and universal agreement scale-level content validity index (S-CVI/UA) were computed. Chance-corrected agreement was assessed using a modified kappa statistic (k*). Items with I-CVI ≥ 0.80 met the minimum quantitative criterion threshold and were considered to be eligible for retention, whereas items I-CVI < 0.80 were excluded according to the predefined rules [38,39,40,41]. In addition to the I-CVI threshold, a contextual applicability criterion—habitual consumption and cultural relevance—was used in the final item retention decision. For each item, I-CVI was calculated as the proportion of experts assigning a rating of 3 or 4 (A/5), where A represents the number of experts endorsing the item as appropriate (3–4). To adjust for chance agreement, the probability of chance agreement (Pc) and the modified kappa (k*) were computed using k* = ((I-CVI) − Pc)/(1 − Pc) [41]. Item-level expert ratings and final decisions for each item are presented in Supplementary Table S1. Following quantitative (I-CVI) and qualitative expert feedback, the index was finalized, with 100 foods organized into nine groups: (1) fruits; (2) vegetables; (3) whole grains; (4) legumes; (5) nuts and oilseeds; (6) fermented foods and products; (7) animal-based foods; (8) functional oils; and (9) spices, herbal teas, and functional beverages. The full component list and scoring table are provided in Table 1.

#### 2.3.3. Scoring of the FunFoCI

The FunFoCI was defined a priori as a formative composite index, where component food items and groups are considered to be contributors that collectively define the construct, rather than reflective indicators of an underlying latent variable. Given the absence of consensus on how to weight functional foods components based on theory or empirical evidence, equal weights were assigned as the main approach to ensure transparency and interpretability. In addition, assigning greater weight to one functional food over another is difficult to justify a priori, since their relative advantages may depend on the specific context of use or health outcome of interest; therefore, an equal-weighting approach is conceptually defensible. Accordingly, it was assumed that each group represents theoretically equivalent dimensions of the index, and the groups were scaled to a 0 to 1 range, regardless of the number of foods they contained.

Because universally accepted cut-offs, standards, or reference intakes for functional foods at the food level as part of the habitual diet are not well established, the FunFoCI uses a within-sample, distribution-based scoring approach, rather than applying absolute thresholds [42,43,44]. FunFoCI scores quantify an individual’s relative position within the analytic sample and are not intended to represent adequacy against external intake recommendations. The scoring system was developed using FFQ data. Instrument-specific cut-offs were then derived separately from FFQ and FR intake distributions within the analytic sample, and the resulting scores were compared as an initial cross-instrument assessment. Because FFQ and FR have different measurement properties, this comparison was designed to evaluate the relative ranking, rather than the absolute intake levels.

The percentile-based scoring approach aims to classify functional foods’ consumption pattern and to quantify relative consumption levels independently of an individual’s total energy. Not adjusting for energy intake was a deliberate methodological choice in this study. Because the primary objective of the study was to develop an index and assess participants’ functional food consumption levels, the primary analyses were performed based on unadjusted consumption data. Furthermore, the sample distribution-based percentile scoring approach may help to relatively balance the energy intake-related bias mechanism by reducing the likelihood that individuals with a higher energy intake automatically receive higher scores.

**Figure 1:**
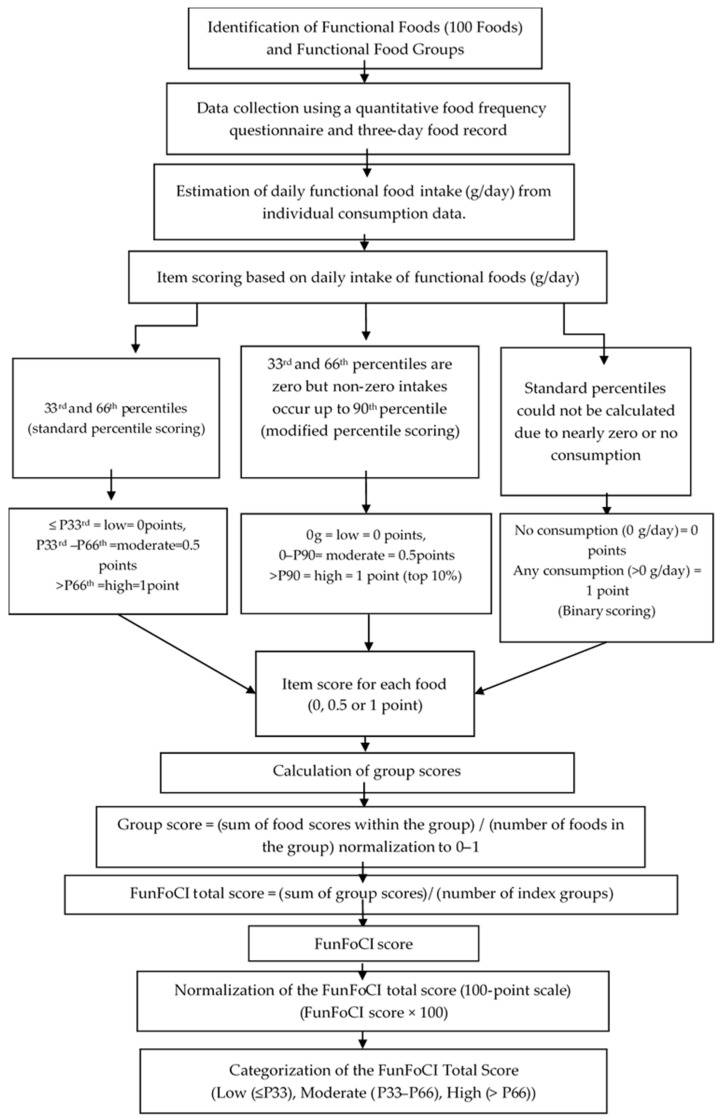
Development of the Functional Food Consumption Index (FunFoCI) and scoring flowchart. P33: 33rd percentile, P66: 66th percentile, P90: 90th percentile. Percentile cut-offs based on study sample distribution.

Sample distribution-based percentile scoring was implemented as follows: the intake of each food item was scored using item-specific percentile cut-offs based on the FFQ-derived intake distribution, with the 33rd and 66th percentiles used as thresholds: 0 points for intake at or below the 33rd percentile, 0.5 points for intake above the 33rd percentile and at or below the 66th percentile, and 1 point for intake above the 66th percentile. For zero-inflated items, where both the 33rd percentile and the 66th percentile were zero and percentile cut-offs were therefore non-informative, a modified rule was applied to preserve discrimination between non-consumers and varying levels among consumers: 0 points for non-consumption (intake = 0), 0.5 points for any non-zero intake at or below the 90th percentile, and 1 point for intake above the 90th percentile. This approach was consistent with prior methods for sparse intake distributions [45] and was intended to better distinguish higher-intake consumers under such conditions. For items with extreme sparsity, where the 33rd, 66th, and 90th percentiles were all zero, binary scoring was applied: 0 points for no consumption and 1 point for any consumption, reflecting the inability of percentile-based cut-offs to meaningfully differentiate intake levels under extreme zero inflation [46]. The scoring flowchart is shown in Figure 1, and item-specific cut-off values are provided in Supplementary Tables S2–S10.

Given the differences in the number of foods across functional food groups, a group- based normalization approach was employed. In order to prevent groups containing a greater number of foods from disproportionately affecting the total index score and to ensure the equal contribution of all groups to the index, each functional food group was normalized to a 0 to 1 scale within the group. For this purpose, group scores were computed as the mean of item scores within each group (ranging from 0 to 1).

The total FunFoCI score was calculated as the mean of the nine group scores (range: 0–1). Thus, each group contributes equally to the total score independently of the number of items within each group. The total score was then multiplied by 100 to yield a 0 to 100 scale, where higher scores indicate higher relative functional food consumption, according to the index. To facilitate interpretation and descriptive reporting, participants were categorized into three groups based on the sample distribution of FunFoCI total scores, corresponding to the lowest 33.3% (low), middle 33.3% (moderate), and highest 33.3% (high). 

### 2.4. Assessment of Diet Quality Indices

DQI-I and HEI-2015 scores were computed from both FFQ and FR data to support validity analyses [43,45,47]. Both indices range from 0 to 100, with higher scores indicating better diet quality [43,45,47]. For DQI-I calculations, reference values were based on Turkish Dietary Guidelines-2022 (TÜBER 2022), Population Reference Intakes (PRI), and Adequate Intake (AI) values [34,48].

### 2.5. Statistical Analysis

Normality was assessed using histograms, Q-Q plots, and the Shapiro–Wilk test. Continuous variables were summarized as mean ± SD for normally distributed data and as the median (IQR) otherwise; categorical variables were *n* (%). Associations between FFQ-based and FR-based FunFoCI scores were evaluated using a correlation coefficient to assess consistency in relative ranking across instruments. Sensitivity analyses were conducted as robustness checks of whether the association between FunFoCI categories and diet quality indices (DQI and HEI) differed by dietary assessment method (FFQ and FR). Content validity was assessed using I-CVI and the scale-level content validity index (S-CVI/Ave). Convergent validity was evaluated by examining associations between FunFoCI scores and diet quality indices (DQI-I and HEI-2015). Spearman’s and Pearson’s correlation methods were used as appropriate. Supplementary evidence for constructed validity, correlations were examined between FunFoCI measures (FFQ-derived and FR-derived), nutrient intakes, and diet quality indices (DQI and HEI; FFQ-derived and FR-derived). Additionally, subgroup comparisons were performed to assess whether FunFoCI total scores differed across participant characteristics. The group differences in continuous variables were performed using one-way ANOVA, based on the variance homogeneity assumption. Tukey’s HSD test was used for post hoc multiple comparisons. Statistical significance was set at *p* < 0.05, and analyses were conducted using SPSS version 31.0 (IBM Corp., Armonk, NY, USA) [49].

## 3. Results

### 3.1. Participant Characteristics and Anthropometric Measures

The study sample comprised 500 adults aged 18–81 years, including 286 women (57.2%) and 214 men (42.8%). The participant characteristics are summarized in Supplementary Table S11. The mean age of the sample was 33.4 ± 13.54 years. In terms of age distribution, participants were predominantly in the 18–24 year group (39.2%). Marital status, education, and occupation variables are summarized in Supplementary Table S11.

Regarding the income-to-expense balance, 44.4% of participants reported that their income was sufficient to cover expenses, 40.2% reported that their income was insufficient to cover expenses, and 15.4% reported that their income exceeded expenses. Smoking status was classified as current, former or never smokers: 31.8% (*n* = 159) were current smokers, 5.0% (*n* = 25) were former smokers, and 63.2% (*n* = 316) were never smokers. The prevalence of self-reported chronic disease was 12.8%, and 10.0% of participants reported regular medication use. Dietary supplement use was reported by 20.0% of participants. Among female participants, 11.9% were postmenopausal. Meal skipping was reported by 67.4% of participants.

The mean of BMI was 25.69 ± 5.20 kg/m^2^. Based on standard BMI categories, 4.6% of participants were underweight, 45.8% were normal weight, 30.4% were overweight, and 19.2% were obese. Anthropometric measurements are summarized in Supplementary Table S12. The prevalence of being overweight was higher in men (39.7%) than women (23.4%), whereas obesity was higher in women (22.7%) than in men (14.5%). An underweight status was uncommon overall but was higher in women (7.0%) than in men (1.4%). For waist circumference risk classification, women showed a higher proportion in the high-risk category (33.2%) compared with men (21.0%). According to WHR classification, a greater proportion of men (49.1%) were categorized as being at risk compared with women (36.7%). For WHtR classification, the proportion in the high-risk group (≥0.60) was slightly higher in women (19.9%) than men (16.4%), whereas women (53.8%) more frequently fell into the normal category (<0.50).

### 3.2. FunFoCI and Diet Quality İndices

The experts evaluated 114 items and the scale-level content validity index were S-CVI/Ave = 0.912 and universal agreement across experts (S-CVI/UA) was 0.877 (Table 2). Item-level CVI values ranged from 0 to 1. Chance-corrected agreement was quantified using modified kappa (mean k* = 0.899 and median k* = 1.00). Fourteen items were excluded during item refinement, including items with low I-CVI values (<0.80) and borderline items that were excluded based on expert feedback. Two items that met the minimum quantitative criterion but yielded borderline indices (I-CVI = 0.80, k* = 0.0763), namely fermented soy products and soybeans, were excluded. The items excluded from the index were cranberry (I-CVI = 0.20), goji berry (I-CVI = 0.20), acai (I-CVI = 0.20), blueberry (I-CVI = 0.20), dragon fruit (pitaya) (I-CVI = 0.00), papaya (I-CVI = 0.00), peas (I-CVI = 0.60), honey (I-CVI = 0.20), royal jelly (I-CVI = 0.00), red wine (I-CVI = 0.00), bay leaf (I-CVI = 0.40), mate leaf (I-CVI = 0.00), fermented soy products (I-CVI = 0.80), and soy beans (I-CVI = 0.80). Although fermented soy products and soybeans met the I-CVI threshold, they were not retained in the final version of the index because of their habitual consumption and cultural applicability. Thus, these two items’ exclusion was based on contextual applicability considerations, rather than on the quantitative content validity criterion alone. The final list comprised 100 functional food items. The remaining items in the index were predominantly staple and commonly consumed foods, and a high level of expert agreement was achieved for these items. Detailed item-level expert ratings and final decisions are presented in Supplementary Table S1.

Regardless of the index score, when the FFQ and FR were examined together across individual functional food items, the highest mean intakes were observed for black tea (FFQ: 301.6 mL/day and FR: 259.1 mL/day), whole wheat and products (FFQ: 159.6 g/day and FR: 111.4 g/day), and tomatoes (FFQ: 108.8 g/day and FR: 85.6 g/day). Black tea predominated in daily consumption (every day 58.0%, at every meal 11.6%); whereas the proportion reporting no consumption of fat-free (skim) milk was 99.4%. In addition, when the percentile-based distribution of FunFoCI scores across the 100 functional food items was examined, commonly consumed staple fruits, vegetables, and traditional foods in the sample, including oranges, mandarins, lemons, apples, bananas, tomatoes, onions, garlic, yogurt, bulgur, and olive oil, were more frequently represented in the upper percentile cut-off (Supplementary Tables S2–S10). In contrast, exotic fruits, certain specialty grains, rarely consumed seeds, specific fermented products, and herbal teas were largely concentrated in the “0” percentile cut-off (Supplementary Tables S2–S10).

**Figure 2:**
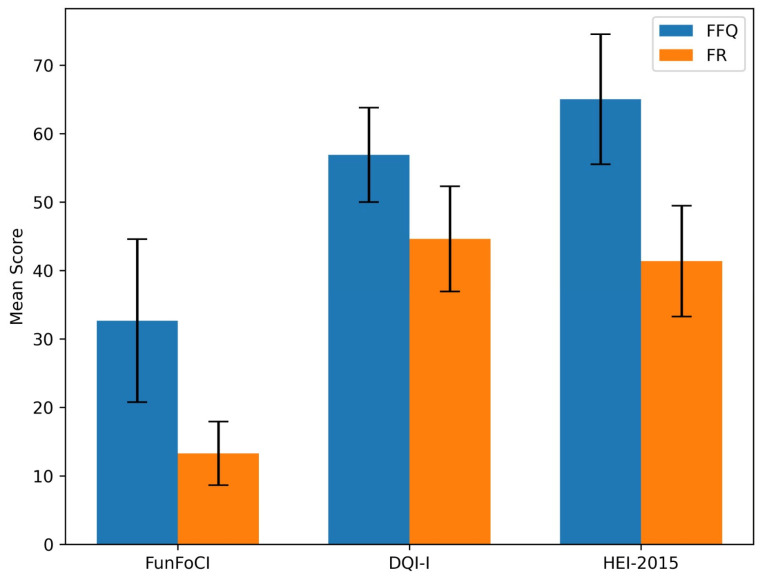
Comparison of mean FunFoCI, DQI-I and HEI scores assessed by FFQ and FR methods. DQI: Diet Quality Index-International and HEI: Healthy Eating Index-2015.

In the study sample, the mean FunFoCI (based on FFQ) total score was 32.68 ± 11.92, and the food record-derived FunFoCI (FunFoCI-FR) score was 13.29 ± 4.65. The mean DQI-I score calculated from the FFQ (DQI-FFQ) was 56.91 ± 6.90, and the mean DQI-I score calculated from the food record (DQI-FR) was 44.63 ± 7.69. The mean HEI-2015 score calculated from the FFQ (HEI-FFQ) was 65.04 ± 9.50, whereas the mean HEI score calculated from the food record (HEI-FR) was 41.38 ± 8.10. These results are presented in Figure 2 and Table 3.

For categorial analyses, the FunFoCI total scores were categorized into percentiles based on sample-derived cut-off (low, moderate, and high). Based on the total score categories, 32.8% of participants (*n* = 164) were classified as low, 33.0% (*n* = 165) as moderate, and 34.2% (*n* = 171) as high functional food consumption (Table 4). When the FunFoCI total score categories were compared with the DQI-FFQ and HEI-FFQ scores, higher DQI-FFQ and HEI-FFQ scores were observed across the increasing FunFoCI scores (*p* < 0.001).

**Figure 3:**
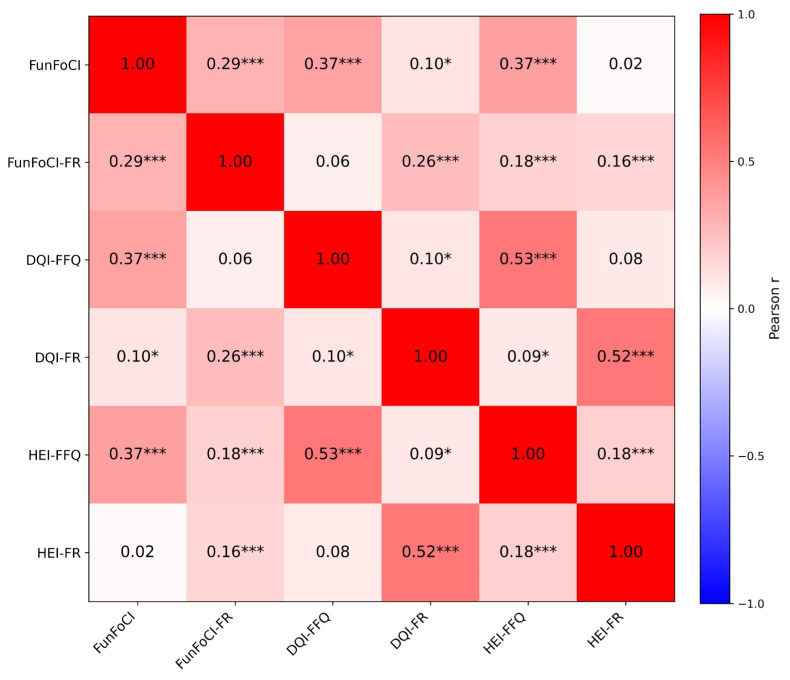
Correlation matrix of FunFoCI, DQI and HEI derived from FFQ and FR. Cells show correlation coefficients (r); color intensity reflects the magnitude of r. Significance * *p* < 0.05 and *** *p* < 0.001. DQI: Diet Quality Index-International, FFQ: Food Frequency Questionnaire, FR: Food Record, and HEI: Healthy Eating Index-2015.

The FunFoCI total scores correlated with DQI-FFQ (r = 0.367; *p* < 0.001) and HEI-FFQ (r = 0.368; *p* < 0.001). FunFoCI total scores were also correlated with FunFoCI-FR (r = 0.294; *p* < 0.001) and with DQI-FR (r = 0.102; *p* = 0.022) (Table 5). No statistically significant correlation was observed between the FunFoCI total scores and HEI-FR (r = 0.020; *p* = 0.664). The correlation matrix is shown in Figure 3. The FunFoCI total scores showed a weak negative correlation with height (r = −0.090; *p* = 0.045). The FunFoCI-FR total scores were not significantly correlated with height (r = 0.028; *p* = 0.529).

As robustness checks, the associations between FunFoCI categories and diet quality indices were compared across assessment methods (Table 6). DQI-I and HEI were computed using FFQ and FR data separately, whereas FunFoCI was derived from FFQ. In the between-group comparisons across FunFoCI consumption levels (low, moderate, high), DQI-FFQ and HEI-FFQ differed significantly across groups (both *p* < 0.001). DQI-FFQ increased from 53.7 ± 8.19 in the low group to 57.9 ± 6.12 and 59.03 ± 4.88 in the moderate and high groups, respectively; post hoc comparisons indicated that the low group had significantly lower DQI-FFQ scores than the moderate and high groups, which did not differ significantly. HEI-FFQ increased from 60.77 ± 10.31 (low) to 65.6 ± 8.10 (moderate) and 68.6 ± 8.29 (high), with post hoc results showing significant differences among all three groups. In contrast, DQI-FR did not differ significantly across FunFoCI levels (*p* = 0.066), and HEI-FR showed no evidence of between-group differences (*p* = 0.986).

The correlations between FunFoCI measures and energy, macro/micro nutrient intakes and diet quality indices (DQI-I, HEI) were examined to support the construct validity; the coefficients are summarized in Supplementary Tables S13 and S14. The relationships between index scores and energy and nutrient intakes that are theoretically expected to be associated with functional food consumption were examined in detail. As shown in Table S13, FFQ-derived FunFoCI scores were positively correlated across nutrients (r = 0.485 to 0.683, all *p* < 0.001), including energy (r = 0.592, *p* < 0.001), protein (r = 0.634, *p* < 0.001), fiber (r = 0.653, *p* < 0.001), and riboflavin (r = 0.683, *p* < 0.001). In contrast, correlations for the FR-derived FunFoCI measure were generally weak (r ≤ 0.163) and mostly non-significant, with small but significant associations observed for only selected nutrients, such as protein (r = 0.099, *p* = 0.027), fat (r = 0.096, *p* = 0.033), fiber (r = 0.097, *p* = 0.030), vitamin A (r = 0.122, *p* = 0.006), carotene (r = 0.138, *p* = 0.002), and vitamin C (r = 0.163, *p* < 0.001). For diet quality indices, DQI-FFQ showed small-to-moderate positive associations with FFQ-derived energy and nutrients (r = 0.105 to 0.418), including energy (r = 0.312, *p* < 0.001), carbohydrate (r = 0.379, *p* < 0.001), fiber (r = 0.410, *p* < 0.001), and potassium (r = 0.418, *p* < 0.001), whereas DQI-FR correlations were negligible overall (r = −0.041 to 0.095), with only carotene (r = 0.095, *p* = 0.034) and riboflavin (r = 0.089, *p* = 0.046) reaching statistical significance. HEI-FFQ was weakly to moderately correlated with selected nutrients, most notably carotene (r = 0.263, *p* < 0.001) and fiber (r = 0.182, *p* < 0.001), while HEI-FR showed no significant associations with energy or nutrient intakes (all *p* > 0.05).

Correlations using FR-derived energy and nutrient intakes are in Table S14. Consistently with method dependence, stronger associations were observed when both the index and intake estimates were derived from the FR. Specifically, FunFoCI-FR correlated positively with FR-derived energy and macronutrients, including energy (r = 0.418, *p* < 0.001), protein (r = 0.371, *p* < 0.001), fat (r = 0.422, *p* < 0.001), carbohydrate (r = 0.321, *p* < 0.001), and fiber (r = 0.356, *p* < 0.001), whereas the FFQ-derived overall FunFoCI score showed generally weak associations with FR-derived intakes. Similarly, DQI-FR showed clearer relationships with FR-derived nutrients, including fiber (r = 0.507, *p* < 0.001) and vitamin C (r = 0.525, *p* < 0.001), while HEI-FR demonstrated expected directions, including positive associations with fiber (r = 0.158, *p* < 0.001) and plant-based protein (r = 0.203, *p* < 0.001).

FunFoCI total scores differed across age groups; the 18–24 year group had the lowest score and differed from the other age groups (*p* < 0.001). No significant difference in FunFoCI total scores was observed by sex (*p* = 0.065). FunFoCI total scores were higher among married participants than single participants (*p* < 0.001). FunFoCI total scores differed by education level, with the highest scores in the postgraduate group (*p* = 0.013), and by occupational status, with the lowest scores being among students and unemployed participants (*p* < 0.001). The FunFoCI total scores did not differ by income level (*p* > 0.05). Never-smokers had higher FunFoCI total scores than current smokers (*p* = 0.007). No significant differences in FunFoCI total scores were observed according to menopausal status, chronic disease, medication use, or supplement use (all *p* > 0.05). Group comparisons are presented in Table 7.

The FunFoCI total scores differed across BMI categories, with the lowest scores in underweight participants and differed from the other groups (*p* < 0.001). No statistically significant differences in the FunFoCI total scores were observed across the waist circumference categories (*p* = 0.075), WHR categories (*p* = 0.105) or WHtR categories (*p* = 0.049). All anthropometric comparisons are presented in Table 8.

The FunFoCI total scores were higher in participants who did not skip meals compared with those who skipped meals (*p* = 0.021). The FunFoCI total scores differed according to the number of snacks consumed per day, with the highest scores among participants consuming three to four snacks (*p* < 0.001).

## 4. Discussion

Functional food consumption has often been assessed using non-standardized or limited approaches, which limits quantitative modeling and complicates the comparability of findings across population subgroups and across studies. To the best of our knowledge, this study represents one of the first attempts to develop a structured, quantitative index of functional food consumption that can be used as a continuous measure in population-based research. Importantly, the primary aim was not to estimate associations with specific health outcomes or to construct an outcome-predictive score, but rather to develop a structured tool for quantifying functional food consumption patterns at the sample level. At the same time, a percentile-based framework may offer a practical advantage for cross-context adaptation by providing a standardized scoring procedure that can be recalibrated for population-specific functional food sets, thereby enabling structured quantitative assessment while preserving local relevance.

In this study, the content validity metrics of the FunFoCI suggest that the retained item set provides appropriate coverage of the target construct within intended context. The pattern of excluded items suggests that expert decisions reflected both functional relevance and contextual considerations, related to habitual consumption in Turkey. Exclusions were clustered in exotic fruits and herbal tea/plant-based products, indicating lower consensus for items with limited consumption or less clear placement within the functional food framework (Supplementary Table S1). The exclusion of red wine and honey was informed by expert judgment and contextual considerations, including uncertainty regarding their placement within the functional food framework and concerns related to alcohol guidance and free sugar content [50,51,52,53]. Additionally, although fermented soy products and soy beans reached the predetermined I-CVI cut-off threshold, they were excluded from the final index based on contextual considerations, as their consumption in Turkey is generally limited and infrequent, which is consistent with national data indicating an annual per capita soy consumption of approximately 0.2 kg [54]. Acceptable thresholds were met under both S-CVI/Ave and S-CVI/UA criteria [40,41] (Table 2). Overall, these results indicate adequate content coverage; however, further evaluation is required to characterize performance in practice and to strengthen evidence for construct and criterion validity [40,41].

FunFoCI scores were moderately and positively correlated with FFQ-derived diet quality indices (DQI-FFQ and HEI-FFQ), and both indices increased across ascending FunFoCI categories. This pattern is consistent with the interpretation that higher functional food consumption co-occurs with a more favorable overall dietary pattern. The correlations were moderate rather than strong, which is plausible because functional foods represent only a subset of dietary choices and may not capture other determinants of diet quality, such as total energy balance, food preparation practices, or intake of ultra-processed foods. Moreover, DQI-I and HEI-2015 are guideline-based and designed to summarize multidimensional adherence to healthy eating recommendations, which may attenuate associations with a single-construct measure such as FunFoCI [43,45,47]. In addition, HEI-2015 is derived from the Dietary Guidelines for Americans (2015 to 2020) and therefore reflects recommendations developed for the U.S. context, which may not fully match every population [55].

An important finding was the decrease in associations when diet quality was derived from food records. While the FunFoCI total scores were correlated with the FunFoCI-FR and showed only a very weak correlation with DQI-FR, no statistically significant correlation was observed with HEI-FR (Figure 2). Because the FFQ and FR cut-offs were derived separately from their respective intake distributions, the resulting scores should be interpreted as method-specific standardized ranks. Accordingly, cross-instrument comparisons primarily reflect the rank-order consistency, rather than the one-to-one comparability of absolute intakes. This approach aligns with the consumption-focused aim of the index: namely, to quantify and classify functional food consumption patterns, rather than to infer absolute intake adequacy. The cross-instrument comparison should be interpreted as an initial assessment; it provides preliminary evidence that the scoring system yields broadly consistent relative classification across two dietary assessment methods, but it does not imply interchangeability or absolute agreement of intake estimates. Consistent with the main findings, supplementary sensitivity analyses (robustness checks) indicated that the FFQ-based and FR-based diet quality indices results diverged considerably (Table 6). Several methodological and contextual factors may contribute to the relatively weak cross-method associations. FFQs and FRs reflect different measurement windows, with FFQs targeting habitual intake and food records capturing the short-term intake over a limited period [56,57]. If functional food consumption is episodic for some items, agreement with short-term records may be reduced. In addition, indices computed from the same assessment method may share method-related variance, which can strengthen within-method association. Finally, food records may be affected by reactivity, under-reporting, and day-to-day variability, potentially reducing correlations with habitual measures [56,57]. Taken together, these findings are consistent with stronger convergence under habitual intake assessment (FFQ) than under short-term recording (FR), although direct method-comparison studies are needed to test this interpretation.

The observed pattern offers supportive, hypothesis-consistent findings that are relevant to construct validity. Overall, FunFoCI showed consistent positive correlations with nutrients that are biologically linked to functional food consumption, including dietary fiber, plant-based protein, carotenoids, vitamin C, folate, potassium, magnesium, and zinc (Supplementary Table S13). These findings support the interpretation that the index reflects a construct related to nutrient density and micronutrient quality. In particular, the stronger associations with fiber, carotenoids, and vitamin C, which are characteristic of plan-derived and antioxidant-rich dietary components [58,59,60], suggest that FunFoCI is capable of capturing dietary patterns enriched in plant-based and functional components.

Importantly, method-comparison results indicated that the magnitude of these associations was partly assessment-modality dependent. When FFQ-derived nutrient intakes were examined (Table S13), the FFQ-derived FunFoCI score showed moderate-to-strong positive correlations across nutrients, whereas correlations involving the FR-derived FunFoCI measure were generally weak. Conversely, when FR-derived nutrient intakes were used (Table S14), stronger correlations were observed within the FR modality, with FunFoCI-FR showing clear positive associations with FR-derived energy and macronutrients, and FR-based diet quality indices demonstrating the expected nutrient relationships. This pattern is compatible with shared-method variance and differences in the error structure of FFQs versus food records, and it also suggests the attenuation of cross-method associations. Because many nutrient intakes covary with total energy intake, the observed correlations may partly reflect the overall intake quantity in addition to dietary composition. Energy-adjusted analyses would help to clarify whether these associations primarily reflect diet quality, rather than overall intake. In addition, because FFQ-derived FunFoCI and FFQ-derived nutrient intakes are based on the same instrument, some degree of method-related covariance is expected and may partly inflate the within-method associations. Given the number of nutrients examined, these results were interpreted with emphasis on the consistency of the observed pattern, rather than statistical significance alone.

The FunFoCI total scores differed by age, marital status, education, and occupational status (Table 7). Participants aged 18 to 24 years had the lowest scores when compared with older age groups. This finding is consistent with reports indicating that young adults may show less health-oriented dietary profiles; for example, lower HEI scores have been observed among young adults, with differences commonly attributed to components such as fruit and vegetable intake and whole-grain consumption [61]. The lower FunFoCI scores in younger adults may reflect differences in food access and food literacy, time constraints related to campus life or early working life, cost-related barriers and more irregular eating routines. Higher scores among married participants and those with postgraduate education may similarly reflect differences in household routines, health-related knowledge, and dietary planning [62]. Evidence from Turkey on the sociodemographic correlations of functional food consumption is heterogeneous [63,64,65,66]. While some studies report associations with education and income [63], others do not observe significant relationships for marital status or income [65]. In this context, the higher FunFoCI scores among married participants in our sample add to the existing evidence and suggest that the role of marital status may be sensitive to sample characteristics and assessment approaches.

FunFoCI scores were also higher among never-smokers than current smokers, which is consistent with the co-occurrence of dietary and lifestyle patterns. Analyses of NHANES data have reported significant differences in HEI scores across smoking categories, and improvements in diet quality have been observed following smoking cessation over time [67]. Similarly, studies using DQI have found that lower diet quality is more common among current smokers than among never smokers [68]. Importantly, these associations should be interpreted as correlational and may be influenced by unmeasured factors such as nutrition knowledge and broader socioeconomic conditions.

FunFoCI scores differed across BMI categories, with the lowest values being in underweight participants, whereas differences by waist circumference and WHR were not significant and the WHtR difference was borderline (Table 8). The borderline WHtR result was not supported by waist circumference or WHR; thus, it was interpreted as weak evidence and treated as non-significant overall. This pattern may reflect that BMI captures the overall weight status, while measures of central adiposity are influenced by multiple factors beyond diet, including physical activity, sleep, stress, and genetic predisposition [69].

Lower FunFoCI scores among participants who skipped meals are consistent with reports linking meal skipping to poorer diet quality. Meal skipping has been associated with lower overall HEI scores and lower intakes of fruits, whole grains, and dairy [70]. Workplace-based studies have also reported that meal skipping is associated with less healthy food choices and lower diet quality [71]. Higher FunFoCI scores among participants consuming 3 to 4 snacks per day may reflect more structured eating patterns and greater dietary variety. However, snack frequency alone is not informative; snack quality is likely the key determinant.

## 5. Conclusions

This study has several strengths, including the structured quantitative assessment of functional food consumption at the population level using the FunFoCI, a relatively large sample, robust expert-based evaluation of item relevance, and the use of more than one dietary assessment approach to contextualize the findings. The interpretability was also supported by relating the FunFoCI scores to two widely used diet quality indices.

Nevertheless, several limitations should also be acknowledged. First, because the study is cross-sectional, the direction of observed associations cannot be determined. Second, most variables were self-reported, which may introduce reporting bias. Third, FFQ-derived and FR-derived scores were interpreted within method-specific scoring frameworks; therefore, consistency across the instrument should be interpreted as pattern-level concordance, rather than the direct comparability or interchangeability of absolute intakes or scores. Fourth, associations, FunFoCI and FFQ-derived and FR-derived indices may be influenced by shared-method variance, and the use of an unadjusted intake means that residual confounding by the total energy intake cannot be fully excluded. Although unadjusted analyses were considered appropriate for initial index development and relative classification, energy-adjusted approaches may strengthen interpretability in future studies, particularly in populations with different energy intake distributions or dietary patterns. Fifth, the scoring framework relied on within-sample, distribution-based cut offs; therefore, FunFoCI primarily reflects the relative consumption ranking within this population and should not be assumed to be directly comparable across populations without recalibration. Sixth, the equal-weighting approach was conceptually defensible for index construction, but it may not capture the outcome-specific biological relevance of different functional food groups. Finally, the definitions, availability, and consumption patterns of functional foods may vary across cultural, regulatory, and food environment contexts, which may limit transportability and require local adaptation and external validation before application in other populations.

Despite these limitations, higher FunFoCI scores were associated with several sociodemographic and lifestyle characteristics and showed the expected relationships with FFQ-derived secondary comparators. Future studies should assess the external validity in different samples and examine the findings by using energy-adjusted analyses and different study designs.

## Figures and Tables

**Table 1:** Component list and scoring of the Functional Food Consumption Index (FunFoCI).

Food Groups	Food Items	Per-Food Score	Group Total Score
Fruits	Strawberry	0 or 0.5 or 1 point	Sum of the scores of all foods within the group/number of foods within the group (24)
	2. Sour cherry/sweet cherry	0 or 0.5 or 1 point	
	3. Blackberry	0 or 0.5 or 1 point	
	4. Raspberry	0 or 0.5 or 1 point	
	5. Black mulberry	0 or 0.5 or 1 point	
	6. Cornelian cherry	0 or 0.5 or 1 point	
	7. Pomegranate	0 or 0.5 or 1 point	
	8. Dried plum (prune)	0 or 0.5 or 1 point	
	9. Dried fig	0 or 0.5 or 1 point	
	10. Dried apricot	0 or 0.5 or 1 point	
	11. Dates	0 or 0.5 or 1 point	
	12. Orange	0 or 0.5 or 1 point	
	13. Grapefruit	0 or 0.5 or 1 point	
	14. Mandarin	0 or 0.5 or 1 point	
	15. Lemon	0 or 0.5 or 1 point	
	16. Persimmon	0 or 0.5 or 1 point	
	17. Banana	0 or 0.5 or 1 point	
	18. Mango	0 or 0.5 or 1 point	
	19. Pineapple	0 or 0.5 or 1 point	
	20. Avocado	0 or 0.5 or 1 point	
	21. Apple	0 or 0.5 or 1 point	
	22. Grape	0 or 0.5 or 1 point	
	23. Watermelon	0 or 0.5 or 1 point	
	24. Melon	0 or 0.5 or 1 point	
Vegetables	1. Broccoli	0 or 0.5 or 1 point	Sum of the scores of all foods within the group/number of foods within the group (23)
	2. Brussels sprouts	0 or 0.5 or 1 point	
	3. White cabbage	0 or 0.5 or 1 point	
	4. Red cabbage	0 or 0.5 or 1 point	
	5. Collard greens	0 or 0.5 or 1 point	
	6. Cauliflower	0 or 0.5 or 1 point	
	7. Leek	0 or 0.5 or 1 point	
	8. Onion	0 or 0.5 or 1 point	
	9. Garlic	0 or 0.5 or 1 point	
	10. Spinach	0 or 0.5 or 1 point	
	11. Purslane	0 or 0.5 or 1 point	
	12. Lettuce/leaf lettuce	0 or 0.5 or 1 point	
	13. Fresh herbs (mint, dill, parsley, etc.)	0 or 0.5 or 1 point	
	14. Arugula/garden cress	0 or 0.5 or 1 point	
	15. Carrot	0 or 0.5 or 1 point	
	16. Beetroot	0 or 0.5 or 1 point	
	17. Celery (stalk or root)	0 or 0.5 or 1 point	
	18. Artichoke	0 or 0.5 or 1 point	
	19. Asparagus	0 or 0.5 or 1 point	
	20. Mushroom	0 or 0.5 or 1 point	
	21. Tomato	0 or 0.5 or 1 point	
	22. Red bell pepper	0 or 0.5 or 1 point	
	23. Green bell pepper	0 or 0.5 or 1 point	
Cereals	1. Whole wheat	0 or 0.5 or 1 point	Sum of the scores of all foods within the group/number of foods within the group (6)
	2. Buckwheat	0 or 0.5 or 1 point	
	3. Rye	0 or 0.5 or 1 point	
	4. Oats	0 or 0.5 or 1 point	
	5. Quinoa	0 or 0.5 or 1 point	
	6. Bulgur	0 or 0.5 or 1 point	
Legumes	1. Red lentil	0 or 0.5 or 1 point	Sum of the scores of all foods within the group/number of foods within the group (7)
	2. Green lentil	0 or 0.5 or 1 point	
	3. Beans (White, borlotti, kidney, etc.)	0 or 0.5 or 1 point	
	4. Chickpea	0 or 0.5 or 1 point	
	5. Black-eyed pea	0 or 0.5 or 1 point	
	6. Fava bean (broad bean)	0 or 0.5 or 1 point	
	7. Mung bean	0 or 0.5 or 1 point	
Nuts and Oilseeds	1. Almond	0 or 0.5 or 1 point	Sum of the scores of all foods within the group/number of foods within the group (10)
	2. Walnut	0 or 0.5 or 1 point	
	3. Hazelnut	0 or 0.5 or 1 point	
	4. Pistachio	0 or 0.5 or 1 point	
	5. Peanut	0 or 0.5 or 1 point	
	6. Chia seed	0 or 0.5 or 1 point	
	7. Flaxseed	0 or 0.5 or 1 point	
	8. Sunflower seed	0 or 0.5 or 1 point	
	9. Pumpkin seed	0 or 0.5 or 1 point	
	10. Sesame	0 or 0.5 or 1 point	
Fermented Foods and Products	1. Kefir	0 or 0.5 or 1 point	Sum of the scores of all foods within the group/number of foods within the group (6)
	2. Probiotic yogurt	0 or 0.5 or 1 point	
	3. Yogurt (homemade, strained, full-fat, low-fat, or fat-free; any non-probiotic type)	0 or 0.5 or 1 point	
	4. Tarhana	0 or 0.5 or 1 point	
	5. Fermented vegetables and products	0 or 0.5 or 1 point	
	6. Şalgam	0 or 0.5 or 1 point	
Animal-Based Foods	1. Fish	0 or 0.5 or 1 point	Sum of the scores of all foods within the group/number of foods within the group (4)
	2. Egg	0 or 0.5 or 1 point	
	3. Seafood and shellfish	0 or 0.5 or 1 point	
	4. Milk (low-fat, reduced-fat, skimmed)	0 or 0.5 or 1 point	
Functional Oils	1. Olive oil	0 or 0.5 or 1 point	Sum of the scores of all foods within the group/number of foods within the group (2)
	2. Hazelnut oil	0 or 0.5 or 1 point	
Spices, Herbal Teas, and Functional Beverages	1. Black pepper	0 or 0.5 or 1 point	Sum of the scores of all foods within the group/number of foods within the group (18)
	2. Ground red pepper/paprika/chili flakes	0 or 0.5 or 1 point	
	3. Thyme	0 or 0.5 or 1 point	
	4. Dried mint	0 or 0.5 or 1 point	
	5. Cumin	0 or 0.5 or 1 point	
	6. Sumac	0 or 0.5 or 1 point	
	7. Turmeric	0 or 0.5 or 1 point	
	8. Ginger	0 or 0.5 or 1 point	
	9. Cinnamon	0 or 0.5 or 1 point	
	10. Cocoa	0 or 0.5 or 1 point	
	11. Sage	0 or 0.5 or 1 point	
	12. Linden	0 or 0.5 or 1 point	
	13. Rosehip	0 or 0.5 or 1 point	
	14. Chamomile	0 or 0.5 or 1 point	
	15. Nigella seed (black seed)	0 or 0.5 or 1 point	
	16. Coffee	0 or 0.5 or 1 point	
	17. Black tea	0 or 0.5 or 1 point	
	18. Green tea	0 or 0.5 or 1 point	
Total Score: (Total score of all groups/number of groups) × 100			0–100

**Table 2:** Summary of content validity indices for the initial candidate functional food items (*n* = 114 items, expert *n* = 5).

Metric	Value
Number of experts (*n*)	5
Number of candidate items	114
S-CVI/Ave	0.9123
S-CVI/UA	0.8772
Mean modified kappa (k*)	0.8994
Median modified kappa (k*)	1.0000
I-CVI (min)	0.0000
I-CVI (max)	1.0000
Distribution of A (0 to 5)	0:5, 1:4, 2:1, 3:2, 4:2, 5:100

S-CVI/Ave: Average scale-level content validity index (mean I-CVI across items), S-CVI/UA: universal agreement scale-level content validity index, I-CVI: item-level content validity index, k*: modified kappa, A: number of experts rating the item as 3 or 4 (relevant/appropriate). The distribution of A (0 to 5) indicates the number of items receiving each possible count of expert relevance ratings. For example, A = 5:100 indicates that 100 items were rated as relevant by all five experts.

**Table 3:** Comparison of mean FunFoCI, DQI-I and HEI scores assessed by FFQ and FR methods.

Indices	$\bar{X}$ ± SD	Min–Max
FunFoCI	32.68 ± 11.92	7.03–78.68
FunFoCI-FR	13.29 ± 4.65	0.55–31.95
DQI-FFQ	56.91 ± 6.90	31.09–82.00
DQI-FR	44.63 ± 7.69	22.52–73.06
HEI-FFQ	65.04 ± 9.50	34.00–87.09
HEI-FR	41.38 ± 8.10	24.00–66.38

Data are presented as mean ± standard deviation ($\bar{X}$ ± SD), minimum–maximum (Min–Max), DQI: Diet Quality Index-International, FFQ: Food Frequency Questionnaire, FR: Food Record, and HEI: Healthy Eating Index-2015.

**Table 4:** FunFoCI and FunFoCI-FR total score categories defined by sample-specific percentiles.

Index	Total Score Category	*n*	%	Cut-Off (Percentile Range)
FunFoCI	Low	164	32.8	≤26.95 (≤P33)
	Moderate	165	33.0	26.96–36.80 (P33–P66)
	High	171	34.2	>36.80 (>P66)
FunFoCI-FR	Low	164	32.8	≤11.20 (≤P33)
	Moderate	164	32.8	11.21–14.65 (P33–P66)
	High	172	34.4	>14.65 (>P66)

P33: 33rd percentile, P66: 66th percentile; percentile cut-offs are based on study sample distribution. FR: Food record.

**Table 5:** Correlations between FunFoCI and the diet quality indices (DQI-I and HEI) total scores derived from FFQ and FR.

Index	Statistic	FunFoCI	FunFoCI-FR	DQI-FFQ	DQI-FR	HEI-FFQ	HEI-FR
FunFoCI	r	1					
	*p*						
FunFoCI-FR	r	0.294 ***	1				
	*p*	<0.001					
DQI-FFQ	r	0.367 ***	0.063	1			
	*p*	<0.001	0.156				
DQI-FR	r	0.102 *	0.261 ***	0.101 *	1		
	*p*	0.022	<0.001	0.024			
HEI- FFQ	r	0.368 ***	0.178 ***	0.529 ***	0.093 *	1	
	*p*	<0.001	<0.001	<0.001	0.037		
HEI-FR	r	0.020	0.159 ***	0.081	0.524 ***	0.183 ***	1
	*p*	0.664	<0.001	0.07	<0.001	<0.001	

Values are correlation coefficients (r) with corresponding *p*-values; Spearman’s and Pearson’s correlation methods were used as appropriate. Statistical significance levels are indicated as * *p* < 0.05 and *** *p* < 0.001. DQI: Diet Quality Index-International, FFQ: Food Frequency Questionnaire, FR: Food Record, and HEI: Healthy Eating Index-2015.

**Table 6:** Comparisons of DQI-I and HEI scores calculated from FFQ and FR across FunFoCI total score categories.

Index	FunFoCI Total Score Category			
	Low $(\bar{X}$ ± SD)	Moderate $(\bar{X}$ ± SD)	High $(\bar{X}$ ± SD)	*p*
DQI-FFQ	53.7 ± 8.19 ^a^	57.9 ± 6.12 ^b^	59.03 ± 4.88 ^b^	<0.001
DQI-FR	43.48 ± 8.52 ^a^	45.08 ± 6.55 ^a^	45.29 ± 7.80 ^a^	0.066
HEI-FFQ	60.77 ± 10.31 ^a^	65.6 ± 8.10 ^b^	68.6 ± 8.29 ^c^	<0.001
HEI-FR	41.31 ± 8.45 ^a^	41.37 ± 7.91 ^a^	41.45 ± 7.98 ^a^	0.986

Values are presented as mean ± standard deviation ($\bar{X}$ ± SD). Differences across three FunFoCI categories were tested using One-Way ANOVA. Pairwise comparisons were performed with Tukey post hoc tests; different superscript letters indicate significant differences (*p* < 0.05). DQI: Diet Quality Index-International, FFQ: Food Frequency Questionnaire, FR: Food Record, and HEI: Healthy Eating Index-2015.

**Table 7:** Comparison of FunFoCI, DQI-FFQ and HEI-FFQ total scores, according to sociodemographic characteristics.

Variable	Group	FunFoCI ($\bar{X}$ ± SD)	*p*	DQI-FFQ ($\bar{X}$ ± SD)	*p*	HEI-FFQ ($\bar{X}$ ± SD)	*p*
Age	18–24	28.53 ± 10.25 a	<0.001	55.83 ± 7.68 a	0.005	62.77 ± 10.12 a	<0.001
	25–34	33.99 ± 14.04 b		56.29 ± 6.09 a		65.41 ± 7.97 ab	
	35–44	36.16 ± 10.79 b		57.95 ± 6.60 ab		66.55 ± 8.70 b	
	45–54	36.38 ± 11.73 b		58.08 ± 5.95 ab		67.31 ± 9.63 b	
	≥55	35.19 ± 10.22 b		59.43 ± 6.26 b		67.64 ± 9.04 b	
Sex	Male	31.54 ± 11.60	0.065	56.10 ± 6.62	0.023	62.78 ± 9.34	<0.001
	Female	33.53 ± 12.11		57.52 ± 7.05		66.73 ± 9.27	
Marital status	Married	36.22 ± 11.55 a	<0.001	58.29 ± 6.25 a	<0.001	67.06 ± 8.99 a	<0.001
	Single	29.63 ± 11.59 b		55.71 ± 7.25 b		63.27 ± 9.71 b	
	Divorced/Widow	36.30 ± 8.79 a		58.53 ± 5.61 a		67.57 ± 6.96 a	
Educational status	Illiterate/Literate	34.01 ± 9.07 a	0.013	59.40 ± 5.99 a	0.005	68.17 ± 6.29 ab	0.017
	Primary School	34.87 ± 13.21 a		59.53 ± 6.47 a		65.83 ± 10.92 ab	
	Middle School	34.91 ± 9.87 a		58.72 ± 7.73 a		69.42 ± 9.06 b	
	High School	32.85 ± 10.82 a		56.34 ± 6.24 b		63.83 ± 8.83 a	
	University/College	31.28 ± 12.26 a		56.16 ± 7.19 b		64.52 ± 9.75 ab	
	Postgraduate	41.05 ± 14.78 b		59.00 ± 4.31 a		66.91 ± 7.98 ab	
Occupation	Student	28.18 ± 10.57 a	<0.001	55.60 ± 7.87 a	0.002	63.50 ± 9.78 ab	0.004
	Self-Employed	35.43 ± 12.97 b		56.72 ± 5.23 ab		64.19 ± 9.01 ab	
	Civil Servant	35.59 ± 12.74 b		56.99 ± 4.89 ab		66.30 ± 8.79 ab	
	Worker	32.37 ± 9.37 ab		57.46 ± 7.93 bc		65.41 ± 10.91 ab	
	Retired	32.18 ± 9.10 ab		58.08 ± 8.16 bc		62.70 ± 10.36 a	
	Homemaker	37.59 ± 10.68 b		59.64 ± 5.58 c		68.73 ± 8.13 b	
	Unemployed	27.43 ± 7.96 a		53.27 ± 7.90 a		62.65 ± 12.56 a	
	Other	34.87 ± 14.48 b		56.66 ± 6.11 ab		64.73 ± 8.15 ab	
Income status	Income Less than Expenses	32.84 ± 11.20	0.549	57.65 ± 6.74	0.084	65.06 ± 9.49	0.997
	Income Equal to Expenses	32.14 ± 12.35		56.17 ± 7.28		65.05 ± 9.56	
	Income Greater than Expenses	33.83 ± 12.54		57.12 ± 6.00		64.97 ± 9.43	
Menopausal status	Yes	34.29 ± 9.49	0.697	59.49 ± 6.30	0.083	69.43 ± 9.58	0.070
	No	33.43 ± 12.43		57.25 ± 7.12		66.37 ± 9.19	
Smoking	Current Smoker	30.25 ± 10.61 a	0.007	55.94 ± 6.67	0.090	63.41 ± 9.85 a	0.019
	Never Smoker	33.91 ± 12.41 b		57.32 ± 6.92		65.94 ± 9.23 b	
	Former Smoker	32.58 ± 11.60 ab		57.94 ± 7.62		64.01 ± 9.34 ab	
Chronic disease	Yes	32.91 ± 12.43	0.867	57.40 ± 5.80	0.539	66.09 ± 9.89	0.346
	No	32.65 ± 11.86		56.84 ± 7.05		64.89 ± 9.44	
Medication use	Yes	32.49 ± 11.43	0.905	57.56 ± 5.55	0.483	65.96 ± 10.20	0.472
	No	32.70 ± 11.99		56.84 ± 7.04		64.94 ± 9.42	
Supplement use	Yes	32.57 ± 11.95	0.915	56.23 ± 6.00	0.268	66.15 ± 9.50	0.187
	No	32.71 ± 11.93		57.08 ± 7.11		64.76 ± 9.49	

Values are presented as mean ± standard deviation ($\bar{X}$ ± SD). Differences were tested using One-Way ANOVA. Pairwise comparisons were determined by a Tukey post hoc test and indicated with different letters. *p* < 0.05 was considered statistically significant.

**Table 8:** Comparison of FunFoCI, DQI-FFQ and HEI-FFQ total scores, according to anthropometric characteristics.

Variable	Classification	FunFoCI ($\bar{X}$ ± SD)	*p*	DQI-FFQ ($\bar{X}$ ± SD)	*p*	HEI-FFQ ($\bar{X}$ ± SD)	*p*
BMI	Underweight	27.44 ± 10.06 a	<0.001	54.53 ± 9.35 a	0.017	64.69 ± 8.80	0.795
	Normal	31.18 ± 11.44 b		56.60 ± 7.09 ab		64.78 ± 9.26	
	Overweight	33.19 ± 11.47 b		56.59 ± 6.85 ab		64.93 ± 10.58	
	Obese	36.70 ± 13.08 c		58.73 ± 5.46 b		65.91 ± 8.44	
Waist circumference	Normal	31.61 ± 12.37	0.075	56.21 ± 7.44 a	0.025	64.77 ± 9.53	0.785
	Risk	33.21 ± 10.68		57.03 ± 5.95 ab		65.46 ± 9.28	
	High risk	34.37 ± 11.68		58.16 ± 6.24 b		65.28 ± 9.63	
Waist-to-hip ratio	Normal	31.94 ± 11.71	0.105	56.76 ± 7.06	0.575	65.50 ± 9.68	0.200
	Risk	33.70 ± 12.16		57.11 ± 6.69		64.40 ± 9.22	
Waist-to-height ratio	Normal (<0.50)	31.39 ± 12.20	0.049	56.33 ± 7.39	0.059	64.97 ± 9.58	0.969
	Risk (0.50–0.59)	33.16 ± 11.27		57.01 ± 6.28		65.20 ± 9.90	
	High risk (≥0.60)	33.74 ± 11.95		58.32 ± 6.33		64.97 ± 8.62	

Values are presented as mean ± standard deviation ($\bar{X}$ ± SD). Differences were tested using One-Way ANOVA. Pairwise comparisons were determined by a Tukey post hoc test and indicated with different letters. *p* < 0.05 was considered statistically significant.

## Data Availability

The data are available from the corresponding author upon reasonable request.

## Supplementary Materials

The following supporting information can be downloaded at: https://www.mdpi.com/article/10.3390/nu18060895/s1, Table S1. Item-level expert ratings and content validity indices for candidate functional food items, and final retention decisions; Table S2. Food-based percentile cut-off and scoring system for the fruits group; Table S3. Food-based percentile cut-offs and scoring system for the vegetables group; Table S4. Food-based percentile cut-offs and scoring system for the whole grains group; Table S5. Food-based percentile cut-offs and scoring system for the legumes group; Table S6. Food-based percentile cut-offs and scoring system for the nuts and oilseeds group.; Table S7. Food-based percentile cut-offs and scoring system for the fermented foods group.; Table S8. Food-based percentile cut-offs and scoring system for the animal-based foods group.; Table S9. Food-based percentile cut-offs and scoring system for functional oils group.; Table S10. Food-based percentile cut-offs and scoring system for spices, herbal teas, and functional beverages.; Table S11. General characteristics of participants (*n* = 500); Table S12. Anthropometric measurements of participants (*n* = 500). Table S13. FFQ-derived energy and nutrient intakes in relation to FunFoCI and diet quality indices (DQI, HEI), by dietary assessment method (FFQ vs. FR); Table S14. FR-derived energy and nutrient intakes in relation to FunFoCI and diet quality indices (DQI, HEI), by dietary assessment method (FFQ vs. FR).

## Abbreviations

The following abbreviations are used in this manuscript:

AI	Adequate intake
BMI	Body mass index
CVI	Content validity index
DQI-FR	Diet quality index-international calculated from food record
DQI-FFQ	Diet quality index-international calculated from food frequency questionnaire
DQI-I	Diet quality index-international
FFQ	Food Frequency Questionnaire
FR	Food record
FunFoCI	Functional Food Consumption Index
FunFoCI-FR	Functional Food Consumption Index calculated from food record
HEI-2015	Healthy Eating Index-2015
HEI-FR	Healthy Eating Index-2015 calculated from food record
HEI-FFQ	Healthy Eating Index-2015 calculated from food frequency questionnaire
I-CVI	Item-level content validity index
k*	Modified kappa statistic
NHANES	National Health and Nutrition Examination Survey
PRI	Population Reference Intakes
S-CVI/Ave	Scale-level content validity index, average method
S-CVI/UA	Scale-level content validity index, universal agreement
SD	Standard deviation
SPSS	Statistical Package for the Social Sciences
TÜBER-2022	Turkish Dietary Guidelines 2022
USD	United States dollar
WHO	World Health Organization
WHR	Waist-to-hip ratio
WHtR	Waist-to-height ratio
