# Supplementary material for: Development and Evaluation of a Functional Food Consumption Index (FunFoCI) in Adults
Source: Nutrients. 2026 Mar 12;18(6):895. doi: 10.3390/nu18060895 (PMC13029523; doi:10.3390/nu18060895)
Supplement: Supplementary file 1 [file nutrients-18-00895-s001.zip › nutrients-4150892-supplementary.pdf]

Supplementary Materials

Supplementary Table S1: Item-level expert ratings and content validity indices for candidate functional food items, and final retention decisions (n = 5 experts).

Candidate Functional Food Items	Expert 1	Expert 2	Expert 3	Expert 4	Expert 5	Number of experts rating as appropriate (3–4) (A)	I-CVI	Pc	k*	Final Decision A ≥ 4 (I-CVI ≥ 0.80)
1. Strawberry	3	3	3	4	3	5	1	0.0312	1	Retained
2. Sour Cherry / cherry	3	3	4	4	4	5	1	0.0312	1	Retained
3. Blackberry	3	3	4	4	4	5	1	0.0312	1	Retained
4. Raspberry	3	3	4	4	4	5	1	0.0312	1	Retained
5. Black mulberry	4	4	4	4	4	5	1	0.0312	1	Retained
6. Cornelian cherry	4	4	4	4	4	5	1	0.0312	1	Retained
7. Pomegranate	4	4	4	4	4	5	1	0.0312	1	Retained
8. Cranberry	2	2	2	3	2	1	0.2	0.1562	0.0519	Excluded
9. Goji berry	1	1	1	3	2	1	0.2	0.1562	0.0519	Excluded
10. Acai	1	1	1	3	2	1	0.2	0.1562	0.0519	Excluded
11. Blueberry	2	2	2	3	2	1	0.2	0.1562	0.0519	Excluded
12. Dried plums (Prunes)	4	4	4	4	4	5	1	0.0312	1	Retained
13. Dried fig	4	4	4	4	4	5	1	0.0312	1	Retained
14. Dried apricot	4	4	4	4	4	5	1	0.0312	1	Retained
15. Dates	4	4	4	4	4	5	1	0.0312	1	Retained
16. Orange	4	4	4	4	4	5	1	0.0312	1	Retained

17.	Grapefruit	4	4	4	4	4	5	1	0.0312	1	Retained
18.	Mandarin	4	4	4	4	4	5	1	0.0312	1	Retained
19.	Lemon	4	4	4	4	4	5	1	0.0312	1	Retained
20.	Persimmon	4	4	4	4	4	5	1	0.0312	1	Retained
21.	Dragon fruit (pitaya)	1	1	1	2	2	0	0	0.0312	-0.0323	Excluded
22.	Banana	3	3	3	4	4	5	1	0.0312	1	Retained
23.	Mango	3	3	3	3	3	5	1	0.0312	1	Retained
24.	Pineapple	3	3	3	4	4	5	1	0.0312	1	Retained
25.	Papaya	1	1	1	1	1	0	0	0.0312	-0.0323	Excluded
26.	Avocado	4	4	4	4	4	5	1	0.0312	1	Retained
27.	Apple	3	3	3	4	4	5	1	0.0312	1	Retained
28.	Grapes	3	3	4	4	4	5	1	0.0312	1	Retained
29.	Watermelon	3	3	4	4	3	5	1	0.0312	1	Retained
30.	Melon	3	3	4	4	3	5	1	0.0312	1	Retained
31.	Broccoli	4	4	4	4	4	5	1	0.0312	1	Retained
32.	Brussels sprouts	3	3	3	3	3	5	1	0.0312	1	Retained
33.	White cabbage	4	4	4	4	4	5	1	0.0312	1	Retained
34.	Red cabbage	4	4	4	4	4	5	1	0.0312	1	Retained
35.	Collard greens	4	4	4	4	4	5	1	0.0312	1	Retained
36.	Cauliflower	4	4	4	4	4	5	1	0.0312	1	Retained
37.	Leek	4	4	4	4	4	5	1	0.0312	1	Retained

38.	Onion	4	4	4	4	4	5	1	0.0312	1	Retained
39.	Garlic	4	4	4	4	4	5	1	0.0312	1	Retained
40.	Spinach	4	4	4	4	4	5	1	0.0312	1	Retained
41.	Purslane	4	4	4	4	4	5	1	0.0312	1	Retained
42.	Lettuce /leaf lettuce	3	3	4	4	4	5	1	0.0312	1	Retained
43.	Fresh herbs (mint, dill, parsley, etc.)	3	3	4	4	4	5	1	0.0312	1	Retained
44.	Arugula / garden cress	3	3	4	4	4	5	1	0.0312	1	Retained
45.	Carrot	4	4	4	4	4	5	1	0.0312	1	Retained
46.	Beetroot	4	4	4	4	4	5	1	0.0312	1	Retained
47.	Celery (stalk or root)	4	4	4	4	4	5	1	0.0312	1	Retained
48.	Artichoke	4	4	4	4	4	5	1	0.0312	1	Retained
49.	Asparagus	4	4	4	4	4	5	1	0.0312	1	Retained
50.	Mushrooms	4	4	4	4	4	5	1	0.0312	1	Retained
51.	Tomato	4	4	4	4	4	5	1	0.0312	1	Retained
52.	Red bell pepper	3	3	4	4	3	5	1	0.0312	1	Retained
53.	Green bell pepper	3	3	3	4	3	5	1	0.0312	1	Retained
54.	Kefir	4	4	4	4	4	5	1	0.0312	1	Retained
55.	Probiotic yogurt	4	4	4	4	4	5	1	0.0312	1	Retained
56.	Yogurt (homemade, strained, full-fat or low-fat)	4	4	4	4	4	5	1	0.0312	1	Retained
57.	Tarhana	4	4	4	4	4	5	1	0.0312	1	Retained

58.	Fermented soy bean products	2	3	3	4	4	4	0.8	0.1562	0.763	Excluded**
59.	Fermented vegetables	3	3	3	3	3	5	1	0.0312	1	Retained
60.	Şalgam	3	3	3	3	3	5	1	0.0312	1	Retained
61.	Whole wheat	4	4	4	4	4	5	1	0.0312	1	Retained
62.	Buckwheat	4	4	4	4	4	5	1	0.0312	1	Retained
63.	Rye	4	4	4	4	4	5	1	0.0312	1	Retained
64.	Oats	4	4	4	4	4	5	1	0.0312	1	Retained
65.	Quinoa	4	4	4	4	4	5	1	0.0312	1	Retained
66.	Bulgur	4	4	4	4	4	5	1	0.0312	1	Retained
67.	Almond	4	4	4	4	4	5	1	0.0312	1	Retained
68.	Walnut	4	4	4	4	4	5	1	0.0312	1	Retained
69.	Hazelnut	4	4	4	4	4	5	1	0.0312	1	Retained
70.	Pistachio	4	4	4	4	4	5	1	0.0312	1	Retained
71.	Peanut	4	4	4	4	4	5	1	0.0312	1	Retained
72.	Chia seeds	4	4	4	4	4	5	1	0.0312	1	Retained
73.	Flaxseed	4	4	4	4	4	5	1	0.0312	1	Retained
74.	Sunflower seeds	3	3	4	4	4	5	1	0.0312	1	Retained
75.	Pumpkin seeds	3	3	4	4	4	5	1	0.0312	1	Retained
76.	Sesame	3	3	4	4	4	5	1	0.0312	1	Retained
77.	Soybeans	3	3	2	4	3	4	0.8	0.1562	0.763	Excluded**
78.	Red Lentils	4	4	4	4	4	5	1	0.0312	1	Retained

79.	Green Lentils	4	4	4	4	4	5	1	0.0312	1	Retained
80.	beans (varieties: white, borlotti, kidney etc.)	4	4	4	4	4	5	1	0.0312	1	Retained
81.	Chickpeas	4	4	4	4	4	5	1	0.0312	1	Retained
82.	Black-eyed peas	4	4	4	4	4	5	1	0.0312	1	Retained
83.	Fava beans (broad bean)	4	4	4	4	4	5	1	0.0312	1	Retained
84.	Peas	2	2	3	3	3	3	0.6	0.3125	0.4182	Excluded
85.	Mung beans	3	3	3	4	4	5	1	0.0312	1	Retained
86.	Fish	4	4	4	4	4	5	1	0.0312	1	Retained
87.	Eggs	3	3	3	4	4	5	1	0.0312	1	Retained
88.	Seafood and shellfish	4	4	4	4	4	5	1	0.0312	1	Retained
89.	Milk (low-fat, semi-skimmed, skimmed)	3	3	3	3	3	5	1	0.0312	1	Retained
90.	Honey	1	2	3	4	4	3	0.6	0.3125	0.4182	Excluded
91.	Cocoa	4	4	4	4	4	5	1	0.0312	1	Retained
92.	Royal jelly	1	1	1	1	1	0	0	0.0312	-0.0323	Excluded
93.	Red Wine	1	1	1	1	1	0	0	0.0312	-0.0323	Excluded
94.	Olive oil	4	4	4	4	4	5	1	0.0312	1	Retained
95.	Hazelnut oil	3	3	3	3	3	5	1	0.0312	1	Retained
96.	Black tea	4	4	4	4	4	5	1	0.0312	1	Retained
97.	Green tea	4	4	4	4	4	5	1	0.0312	1	Retained
98.	Coffee	4	4	4	4	4	5	1	0.0312	1	Retained
99.	Cumin	4	4	4	4	4	5	1	0.0312	1	Retained

100. Black pepper	4	4	4	4	4	5	1	0.0312	1	Retained
101. Ground red pepper (paprika/chili)	4	4	4	4	4	5	1	0.0312	1	Retained
102. Thyme / Oregano	4	4	4	4	4	5	1	0.0312	1	Retained
103. Sumac	3	3	3	4	4	5	1	0.0312	1	Retained
104. Ginger	4	4	4	4	4	5	1	0.0312	1	Retained
105. Bay leaf	2	2	2	3	3	2	0.4	0.3125	0.1273	Excluded
106. Cinnamon	4	4	4	4	4	5	1	0.0312	1	Retained
107. Turmeric	4	4	4	4	4	5	1	0.0312	1	Retained
108. Black seed (Nigella seed)	3	3	3	3	3	5	1	0.0312	1	Retained
109. Linden tea	4	4	4	4	4	5	1	0.0312	1	Retained
110. Dried Mint	3	3	3	4	4	5	1	0.0312	1	Retained
111. Sage tea	4	4	4	4	4	5	1	0.0312	1	Retained
112. Chamomile	3	3	3	3	3	5	1	0.0312	1	Retained
113. Rosehip tea	3	3	4	4	4	5	1	0.0312	1	Retained
114. Yerba mate (mate leaf)	1	1	1	1	1	0	0	0.0312	-0.0323	Excluded

A (3–4): number of experts rating the item as 3 or 4 (considered "appropriate"); maximum rating = 5., I-CVI: Item-Level Content Validity Index, calculated as A/5, Pc: probability of chance agreement for A out of 5 experts rating the item as 3 or 4, (k*): modified kappa statistic adjusting I-CVI for chance agreement, computed as k* = (I-CVI − Pc) / (1 − Pc). ****:** Items with A ≥ 4 (I-CVI ≥ 0.80) met the minimum quantitative criterion for retention; however, following review to ensure conceptual alignment with contextual applicability, a small number of eligible items were excluded, yielding the final retained list. Decision categories: Retained, included in the final list; Excluded, removed from the final list, Rating scale: 1=not representative, 2= representative with major revision, 3= representative, 4= highly representative (4-point scale).

Food-Based Percentile Cut-offs and Scoring System for the Functional Food Consumption Index (FunFoCI)

Foods included in Functional Food Consumption Index (FunFoCI) were scored according to sample-specific 33rd and 66th percentile cut-off values. For foods with infrequent consumption and zero percentile values, a modified percentile approach was applied considering the zero-inflated nature of the distribution, and the 90th percentile was accepted as the high consumption threshold for these foods. Accordingly, no consumption was evaluated as 0 points, consumption below and at the 90th percentile as 0.5 points, and consumption above the 90th percentile as 1 point. For foods where the 33rd, 66th, and 90th percentile values were all zero, a binary scoring system was applied because quantitative percentile-based classification was not possible; absence of consumption was scored as 0 points, and presence of consumption at any level was scored as 1 point.

Supplementary Table S2. Food-Based Percentile Cut-offs and Scoring System for the Fruits Group

Food	33rd Percentile	66th Percentile	0 Points	0.5 Points	1 Points	Scoring Type
Strawberry (g)	4,82	21,40	≤ P33	P33-P66	>P66	Standard percentile
Sour cherry/sweet Cherry (g)	3,30	21,40	≤ P33	P33-P66	>P66	Standard percentile
Blackberry (g)	0,00	2,19	≤ P33	P33-P66	>P66	Standard percentile
Raspberry (g)	0,00	0,00	0 g	> 0 –90%	>P90(top 10%)	Modified percentile
Black mulberry (g)	0,00	1,65	≤ P33	P33-P66	>P66	Standard percentile
Cornelian cherry (g)	0,00	0,00	0 g	>0 –P90	>P90 (top 10%)	Modified percentile
Pomegranate (g)	5,72	32,10	≤ P33	P33-P66	>P66	Standard percentile
Dried plum (prune) (g)	0,00	0,00	0 g	>0 –P90	>P90 (top 10%)	Modified percentile
Dried fig (g)	0,00	1,98	≤ P33	P33-P66	>P66	Standard percentile
Dates (g)	0,00	1,49	≤ P33	P33-P66	>P66	Standard percentile
Dried apricot (g)	1,43	31,74	≤ P33	P33-P66	>P66	Standard percentile
Orange (g)	29,96	79,35	≤ P33	P33-P66	>P66	Standard percentile
Grapefruit (g)	0,00	2,31	≤ P33	P33-P66	>P66	Standard percentile
Mandarin (g)	13,91	51,09	≤ P33	P33-P66	>P66	Standard percentile
Lemon (g)	12,84	47,16	≤ P33	P33-P66	>P66	Standard percentile
Persimmon (g)	0,00	6,60	≤ P33	P33-P66	>P66	Standard percentile
Banana (g)	15,87	42,80	≤ P33	P33-P66	>P66	Standard percentile
Mango (g)	0,00	0,00	0 g	0g	>0 g	Binary
Pineapple (g)	0,00	0,00	0 g	>0 –P90	>P90(top 10%)	Modified percentile
Avocado (g)	0,00	0,00	0 g	>0 –P90	>P90(top 10%)	Modified percentile

Apple (g)	32,10	79,35	≤ P33	P33-P66	>P66	Standard percentile
Grape (g)	6,82	42,80	≤ P33	P33-P66	>P66	Standard percentile
Watermelon (g)	14,52	105,8	≤ P33	P33-P66	>P66	Standard percentile
Melon (g)	13,2	47,08	≤ P33	P33-P66	>P66	Standard percentile

For Raspberry (9.873 g), Cranberry (4.95 g), Dried Plum (3.96 g), Mango (0 g), Pineapple (6.6 g), and Avocado (15.84 g), above the 90th percentile, representing the top 10%, was considered the high consumption threshold. P33: 33rd percentile, P66: 66th percentile, percentiles cut-offs based on study sample distribution.

Supplementary Table S3. Food-Based Percentile Cut-offs and Scoring System for the Vegetables Group

Food	33rd Percentile	66th Percentile	0 Points	0,5 Points	1 Points	Scoring Type
Broccoli (g)	0,00	6,04	≤ P33	P33-P66	>P66	Standard percentile
Brussels sprouts (g)	0,00	0,00	0 g	>0 –P90	>P90(top 10%)	Modified percentile
White cabbage (g)	0,00	7,69	≤ P33	P33-P66	>P66	Standard percentile
Red cabbage (g)	0,00	7,69	≤ P33	P33-P66	>P66	Standard percentile
Collard greens (g)	0,00	6,60	≤ P33	P33-P66	>P66	Standard percentile
Cauliflower (g)	0,00	9,90	≤ P33	P33-P66	>P66	Standard percentile
Leek (g)	0,00	6,60	≤ P33	P33-P66	>P66	Standard percentile
Onion (g)	36,63	100,00	≤ P33	P33-P66	>P66	Standard percentile
Garlic (g)	0,59	3,00	≤ P33	P33-P66	>P66	Standard percentile
Spinach (g)	2,48	9,90	≤ P33	P33-P66	>P66	Standard percentile
Purslane (g)	0,00	4,95	≤ P33	P33-P66	>P66	Standard percentile
Lettuce/leafy lettuce (g)	16,05	39,68	≤ P33	P33-P66	>P66	Standard percentile
Fresh herbs (mint, dill, and parsley)	5,31	25,64	≤ P33	P33-P66	>P66	Standard percentile
Arugula (g)	4,82	21,40	≤ P33	P33-P66	>P66	Standard percentile
Sorrel (g)	0,00	0,00	≤ P33	P33-P66	>P66	Standard percentile
Carrot (g)	8,79	39,68	≤ P33	P33-P66	>P66	Standard percentile
Beetroot (g)	0,00	0,00	0 g	>0 –P90	>P90(top 10%)	Modified percentile
Celery (g)	0,00	0,00	0 g	>0 –P90	>P90(top 10%)	Modified percentile
Artichoke (g)	0,00	0,00	0 g	>0 –P90	>P90(top 10%)	Modified percentile
Asparagus (g)	0,00	0,00	0 g	0g	>0 g	Binary
Mushroom (g)	0,00	6,93	0 g	>0 –P90	>P90(top 10%)	Modified percentile
Red bell pepper (g)	3,30	21,40	≤ P33	P33-P66	>P66	Standard percentile
Green bell pepper (g)	7,49	22,47	≤ P33	P33-P66	>P66	Standard percentile
Tomato (g)	68,77	117,90	≤ P33	P33-P66	>P66	Standard percentile

For Brussels sprouts (4.95 g), sorrel (14.98 g), beetroot (13.20 g), celery (1.49 g), artichoke (2.28 g), above the 90th percentile, representing the top 10%, was considered the high consumption threshold. P33: 33rd percentile, P66: 66th percentile, percentiles cut-offs based on study sample distribution.

Supplementary Table S4. Food-Based Percentile Cut-offs and Scoring System for the Whole Grains Group

Food	33rd Percentile	66th Percentile	0 Points	0,5 Points	1 Points	Scoring Type
Whole wheat (g)	52,90	150,00	≤ P33	P33-P66	>P66	Standard percentile
Oat (g)	0,00	0,00	0 g	>0 –P90	>P90(top 10%)	Modified percentile
Rye (g)	0,00	0,00	0 g	>0 –P90	>P90(top 10%)	Modified percentile
Bulgur (g)	4,28	15,72	≤ P33	P33-P66	>P66	Standard percentile
Buckwheat (g)	0,00	0,00	0 g	0g	>0 g	Binary
Quinoa (g)	0,00	0,00	0 g	0g	>0 g	Binary

For rye (1.65 g) and oats (5.31 g) above the 90th percentile, representing the top 10%, was accepted as the high consumption threshold. P33: 33rd percentile, P66: 66th percentile, percentiles cut-offs based on study sample distribution.

Supplementary Table S5. Food-Based Percentile Cut-offs and Scoring System for the Legumes Group

Food	33rd Percentile	66th Percentile	0 Points	0,5 Points	1 Points	Scoring Type
Red lentil (g)	6,42	10,58	≤ P33	P33-P66	>P66	Standard percentile
Green lentil (g)	1,98	7,49	≤ P33	P33-P66	>P66	Standard percentile
Beans (g)	3,30	10,70	≤ P33	P33-P66	>P66	Standard percentile
Chickpea (g)	2,31	10,70	≤ P33	P33-P66	>P66	Standard percentile
Black-eyed pea (g)	0,00	0,00	0 g	>0 –P90	>P90(top 10%)	Modified percentile
Broad bean (fava beans) (g)	0,00	0,00	0 g	>0 –P90	>P90(top 10%)	Modified percentile
Mung bean (g)	0,00	0,00	0 g	0g	>0 g	Binary

For black-eyed peas (1.65 g), broad beans (6.60 g) above the 90th percentile, representing the top 10%, was considered the high consumption threshold. P33: 33rd percentile, P66: 66th percentile, percentiles cut-offs based on study sample distribution.

Supplementary Table S6. Food-Based Percentile Cut-offs and Scoring System for the Nuts and Oilseeds Group.

Food	33rd Percentile	66th Percentile	0 Points	0,5 Points	1 Points	Scoring Type
Almond (g)	0,83	5,35	≤ P33	P33-P66	>P66	Standard percentile
Walnut (g)	1,65	6,42	≤ P33	P33-P66	>P66	Standard percentile
Hazelnut (g)	1,65	6,42	≤ P33	P33-P66	>P66	Standard percentile
Pistachio (g)	0,83	5,35	≤ P33	P33-P66	>P66	Standard percentile
Peanut (g)	0,33	2,47	≤ P33	P33-P66	>P66	Standard percentile
Chia seed (g)	0,00	0,00	0 g	>0 –P90	>P90(top 10%)	Modified percentile

Sunflower seed (g)	0,66	4,28	≤ P33	P33-P66	>P66	Standard percentile
Pumpkin seed (g) (g)	0,00	0,83	≤ P33	P33-P66	>P66	Standard percentile
Flaxseed (g)	0,00	0,00	0 g	0g	>0 g	Binary
Sesame (g)	0,00	0,10	≤ P33	P33-P66	>P66	Standard percentile

Chia seed (0.33 g) above the 90th percentile, representing the top 10%, was considered the high consumption threshold. P33: 33rd percentile, P66: 66th percentile, percentiles cut-offs based on study sample distribution.

Supplementary Table S7. Food-Based Percentile Cut-offs and Scoring System for the Fermented Foods Group.

Food	33rd Percentile	66th Percentile	0 Points	0,5 Points	1 Points	Scoring Type
Kefir (g)	0,00	0,00	0 g	>0 –P90	>P90(top 10%)	Modified percentile
Probiotic yogurt (g)	0,00	0,00	0 g	>0 –P90	>P90(top 10%)	Modified percentile
Yogurt (g)	32,10	100,00	≤ P33	P33-P66	>P66	Standard percentile
Tarhana (g)	0,00	1,98	≤ P33	P33-P66	>P66	Standard percentile
Fermented vegetables (g)	0,00	0,00	0 g	0g	>0 g	Binary
Şalgam (mL)	0,00	0,00	0 g	>0 –P90	>P90(top 10%)	Modified percentile

P33: 33rd percentile, P66: 66th percentile, percentiles cut-offs based on study sample distribution.

Supplementary Table S8. Food-Based Percentile Cut-offs and Scoring System for the Animal-Based Foods Group.

Food	33rd Percentile	66th Percentile	0 Points	0,5 Points	1 Points	Scoring Type
Fish (g)	4,95	10,56	≤ P33	P33-P66	>P66	Standard percentile
Egg (g)	29,96	55,02	≤ P33	P33-P66	>P66	Standard percentile
Seafood and shellfish (g)	0,00	4,95	≤ P33	P33-P66	>P66	Standard percentile
Milk (low-fat/ reduced-fat/ skimmed) (g)	0,00	0,00	0 g	0g	>0 g	Binary

P33: 33rd percentile, P66: 66th percentile, percentiles cut-offs based on study sample distribution.

Supplementary Table S9. Food-Based Percentile Cut-offs and Scoring System for Functional Oils Group.

Food	33rd Percentile	66th Percentile	0 Points	0,5 Points	1 Points	Scoring Type
Olive oil (g)	1,32	10,58	≤ P33	P33-P66	>P66	Standard percentile
Hazelnut oil (g)	0,00	0,00	0 g	0g	>0 g	Binary

P33: 33rd percentile, P66: 66th percentile, percentiles cut-offs based on study sample distribution.

Supplementary Table S10. Food-Based Percentile Cut-offs and Scoring System for Spices, Herbal Teas, and Functional Beverages.

Food	33rd Percentile	66th Percentile	0 Points	0,5 Points	1 Points	Scoring Type
Black tea (mL)	157,20	250,00	≤ P33	P33-P66	>P66	Standard percentile
Green tea (mL)	0,00	6,60	≤ P33	P33-P66	>P66	Standard percentile
Coffee (mL)	7,78	51,09	≤ P33	P33-P66	>P66	Standard percentile
Cocoa (g)	0,17	0,99	≤ P33	P33-P66	>P66	Standard percentile
Sage (g)	0,00	0,00	0 g	>0 –P90	>P90(top 10%)	Modified percentile
Linden (g)	0,00	8,25	≤ P33	P33-P66	>P66	Standard percentile
Rosehip tea (mL)	0,00	0,00	0 g	>0 –P90	>P90(top 10%)	Modified percentile
Chamomile tea (mL)	0,00	0,00	0 g	>0 –P90	>P90(top 10%)	Modified percentile
Turmeric (g)	0,00	0,00	0 g	>0 –P90	>P90(top 10%)	Modified percentile
Black pepper (g)	0,20	0,50	≤ P33	P33-P66	>P66	Standard percentile
Red pepper ground/flakes (g)	0,00	0,00	0 g	>0 –P90	>P90(top 10%)	Modified percentile
Thyme (g)	0,02	0,11	≤ P33	P33-P66	>P66	Standard percentile
Cinnamon (g)	0,00	0,02	≤ P33	P33-P66	>P66	Standard percentile
Ginger (g)	0,00	0,00	0 g	>0 –P90	>P90(top 10%)	Modified percentile
Cumin (g)	0,00	0,11	≤ P33	P33-P66	>P66	Standard percentile
Sumac (g)	0,00	0,04	≤ P33	P33-P66	>P66	Standard percentile
Nigella seeds (g)	0,00	0,08	≤ P33	P33-P66	>P66	Standard percentile
Dried mint (g)	0,03	0,26	≤ P33	P33-P66	>P66	Standard percentile

for sage (13.20 g), rosehip tea (21.78 g), chamomile tea (6.60 g), turmeric (0.11 g), red pepper (6.60 g), and ginger (0.03 g) above the 90th percentile, representing the top 10%, was considered the high consumption threshold. P33: 33rd percentile, P66: 66th percentile, percentiles cut-offs based on study sample distribution.

Supplementary Table S11. General characteristics of Participants (n = 500)

		n	%
**Age (group)**	18–24 years	196	39.2
	25–34 years	104	20.8
	35–44 years	78	15.6
	45–54 years	84	16.8
	≥55 years	38	7.6
	$\bar{X}$ ± SD =33.54 ± 13.54, median (Min.-Max.) = 29 (18–81)		
**Sex**	Male	214	42.8
	Female	286	57.2
**Marital status**	Married	212	42.4
	Single	269	53.8
	Divorced/Widowed	19	3.8
**Educational status**	Illiterate/Literate	21	4.2
	Primary school	43	8.6
	Middle school	36	7.2
	High school	128	25.6
	University/College	256	51.2
	Postgraduate	16	3.2
**Occupation**	Student	172	34.4
	Self-employed	56	11.2
	Civil servant	62	12.4
	Worker	48	9.6
	Retired	21	4.2
	Housewife	82	16.4
	Unemployed	9	1.8
	Other	50	10.0
**Income status**	Income less than expenses	201	40.2
	Income equal to expenses	222	44.4
	Income greater than expenses	77	15.4
**Menopausal status** *	Yes	34	11.9
	No	252	88.1
**Smoking status**	Current Smoker	159	31.8
	Never Smoker	316	63.2
	Former smoker	25	5.0
**Chronic disease**	Yes	64	12.8
	No	436	87.2
**Medication use**	Yes	50	10.0
	No	450	90.0
**Supplement use**	Yes	101	20.2
	No	399	79.8
**Skipping meals**	Yes	337	67.4
	No	163	32.6
**Number of main meals**	≤1 main meal	13	2.6
	2 main meals	259	51.8
	≥3 main meals	228	45.6
**Number of snacks**	No snacks	94	18.8
	1 snack	189	37.8
	2 snacks	170	34.0
	3 - 4 snacks	47	9.4

Data are presented as mean ± standard deviation ($\bar{X}$ ± SD) and median (minimum–maximum).
*Menopausal status was evaluated only for female participants (n = 286).

Supplementary Table S12. Anthropometric Measurements of Participants (n = 500)

Anthropometric measurements	X̄ ± SD	Median (Min.-Max.)
Body weight (kg)	73.81 ± 26.64	72 (37–552)
Height (cm)	168.28 ± 9.46	167 (145–198)
Body Mass Index (kg/m²)	25.69 ± 5.20	24.96 (15.01–45.76)
Waist circumference (cm)	86.38 ± 22.85	84 (2–405)
Hip circumference (cm)	101.00 ± 17.89	100 (10–225)
Waist-to-hip ratio	0.89 ± 0.54	0.85 (0.02–9.60)
Waist-to-height ratio	0.51 ± 0.14	0.49 (0.01–2.45)
	**Male n (%)**	**Female n (%)**
**BMI classification**		
Underweight (<18.5)	3 (1.4)	20 (7.0)
Normal weight (18.5–24.99)	95 (44.4)	134 (46.9)
Overweight (25.0–29.99)	85 (39.7)	67 (23.4)
Obese (≥30.0)	31 (14.5)	65 (22.7)
**Waist circumference**		
Normal (M: <94). (F: <80)	125 (58.4)	142 (49.7)
Risk (M: ≥94–102). (F: ≥80–88)	44 (20.6)	49 (17.1)
Increased risk (M: ≥102). (F: ≥88)	45 (21.0)	95 (33.2)
**Waist-to-hip ratio**		
Normal (M: <0.90). (F: <0.85)	109 (50.9)	181 (63.3)
At risk (M: ≥0.90). (F: ≥0.85)	105 (49.1)	105 (36.7)
**Waist-to-height ratio**		
Normal (<0.50)	99 (46.3)	154 (53.8)
Increased risk (0.50–0.59)	80 (37.4)	75 (26.2)
High risk (≥0.60)	35 (16.4)	57 (19.9)

Mean ± standard deviation (X̄ ± SD), minimum–maximum (Min–Max)

Supplementary Table S13. FFQ-derived energy and nutrient intakes in relation to FunFoCI and diet quality indices (DQI, HEI), by dietary assessment method (FFQ vs FR).

FFQ-Variables	Statistics	FunFoCI	FunFoCI-FR	DQI-FFQ	DQI-FR	HEI-FFQ	HEI-FR
Energy (kcal/day)	r	0,592***	0,056	0,312***	0,024	-0,026	-0,024
	p	<0,001	0,215	<0,001	0,597	0,562	0,587
Protein (g/day)	r	0,634***	0,099*	0,334***	0,085	0,054	0,016
	p	<0,001	0,027	<0,001	0,057	0,231	0,721
Fat (g/day)	r	0,576***	0,096*	0,112*	0,006	0,005	0,008
	p	<0,001	0,033	0,012	0,902	0,91	0,857
Carbohydrate (g/day)	r	0,489***	0,010	,379***	0,017	-0,061	-0,050
	p	<0,001	0,825	<0,001	0,713	0,172	0,267
Fiber (g/day)	r	,653***	0,097*	0,410***	0,064	0,182***	0,025
	p	<0,001	0,030	<0,001	0,155	<0,001	0,582
Plant-based protein (g/day)	r	0,636***	0,064	,405***	0,068	0,098*	0,004
	p	<0,001	0,154	<0,001	0,128	0,029	0,931
Vit. A (µg/day)	r	0,559***	0,122**	0,223***	0,082	0,136**	-0,018
	p	<0,001	0,006	<0,001	0,067	0,002	0,694
Carotene (mg/day)	r	0,645***	0,138**	0,339***	0,095*	0,263***	0,049
	p	<0,001	0,002	<0,001	0,034	<0,001	0,270
Vit. E (mg α-TE/day)	r	0,542***	0,072	0,229***	-0,015	0,181***	0,019
	p	<0,001	0,106	<0,001	0,741	<0,001	0,678
Thiamin (mg/day)	r	0,643***	0,054	0,384***	0,054	0,105*	-0,010
	p	<0,001	0,227	<0,001	0,227	0,019	0,829
Riboflavin (mg/day)	r	0,683***	0,126**	0,304***	0,089*	0,069	0,002
	p	<0,001	0,005	<,001	0,046	0,125	0,959
Pyridoxine (mg/day)	r	0,613***	0,071	0,376***	0,050	0,130**	0,005
	p	<0,001	0,115	<,001	0,269	0,004	0,919
Folate (µg/day)	r	0,603***	0,071	0,397***	0,065	0,167***	-0,007
	p	<0,001	0,112	<0,001	0,145	<0,001	0,880
Vit. C (mg/day)	r	0,557***	0,163***	0,250***	0,081	0,170***	0,022
	p	<0,001	<0,001	<0,001	0,07	<0,001	0,623
Potassium (mg/day)	r	0,649***	0,086	0,418***	0,055	0,168***	0,009
	p	<0,001	0,054	<0,001	0,219	<0,001	0,844
Calcium (mg/day)	r	0,643***	0,095*	0,282***	0,067	0,059	-0,002
	p	<0,001	0,033	<0,001	0,134	0,191	0,971
Magnesium (mg/day)	r	0,671***	0,080	0,359***	0,036	0,169***	0,020
	p	<0,001	0,074	<0,001	0,425	<0,001	0,659
Phosphorus (mg/day)	r	0,673***	0,097*	0,346***	0,049	0,094*	0,008
	p	<0,001	0,03	<0,001	0,271	0,035	0,854
Iron (mg/day)	r	0,612***	0,053	0,321***	0,064	0,099*	0,047
	p	<0,001	0,241	<0,001	0,151	0,027	0,297
Zinc (mg/day)	r	0,652***	0,101*	0,324***	0,079	0,079	0,028
	p	<0,001	0,024	<0,001	0,077	0,076	0,526
Niacin (mg/day)	r	0,590***	0,077	,331***	0,062	0,052	0,008
	p	<0,001	0,084	<0,001	0,168	0,246	0,856
PUFA (g/day)	r	0,485***	0,058	0,156***	-0,041	0,113*	0,036
	p	<0,001	0,196	<0,001	0,359	0,012	0,419
MUFA (g/day)	r	0,563***	0,105*	0,105*	0,009	0,087	0,018
	p	<0,001	0,019	0,019	0,841	0,051	0,69

Values are correlation coefficients (r) with corresponding p-values; Spearman's and Pearson's correlation methods were used as appropriate. Statistical significance was indicated as *p<0.05, **p<0.01, and ***p<0.001. PUFA: polyunsaturated fatty acids; MUFA: monounsaturated fatty acids. α-TE: α-tocopherol equivalents.

Supplementary Table S14. FR-derived energy and nutrient intakes in relation to FunFoCI and diet quality indices (DQI, HEI), by dietary assessment method (FFQ vs FR).

FR-Variables	Statistics	FunFoCI	FunFoCI-FR	DQI-FFQ	DQI-FR	HEI-FFQ	HEI-FR
Energy (kcal/day)	r	0,037	0,418***	-0,086	0,222***	-0,139**	-0,061
	p	0,414	<0,001	0,054	<0,001	0,002	0,171
Protein (g/day)	r	0,001	0,371***	-0,065	0,331***	-0,063	0,065
	p	0,986	<0,001	0,15	<0,001	0,162	0,146
Fat (g/day)	r	0,087	0,422***	-0,044	-0,023	-0,088*	-0,177***
	p	0,052	<0,001	0,328	0,607	0,049	<0,001
Carbohydrate (g/day)	r	-0,011	0,321***	-0,105*	0,318***	-0,173***	0,006
	p	0,801	<0,001	0,019	<0,001	<0,001	0,892
Fiber (g/day)	r	0,031	0,356***	0,017	0,507***	0,010	0,158***
	p	0,490	<0,001	0,709	<0,001	0,826	<0,001
Plant-based protein (g/day)	r	0,002	0,288***	-0,009	0,122**	-0,047	0,203***
	p	0,958	<0,001	0,834	0,006	0,297	<0,001
Vit. A (μg/day)	r	0,069	0,162***	0,05	0,169***	0,014	0,009
	p	0,126	<0,001	0,267	<0,001	0,755	0,833
Carotene (mg/day)	r	0,034	0,239***	0,036	0,437***	0,064	0,173***
	p	0,451	<0,001	0,422	<0,001	0,151	<0,001
Vit. E (mg α-TE/day)	r	-0,064	0,191***	-0,037	0,342***	-0,038	0,195***
	p	0,152	<0,001	0,407	<0,001	0,398	<0,001
Thiamin (mg/day)	r	0,032	0,388***	-0,018	0,435***	-0,001	0,152***
	p	0,474	<0,001	0,689	<0,001	0,989	<0,001
Riboflavin (mg/day)	r	0,114*	0,424***	-0,010	0,243***	-0,064	-0,083
	p	0,01	<0,001	0,822	<0,001	0,155	0,063
Pyridoxine (mg/day)	r	-0,024	0,264***	-0,021	0,423***	-0,056	0,174***
	p	0,592	<0,001	0,639	<0,001	0,210	<0,001
Folate (μg/day)	r	0,041	0,300***	0,050	0,420***	0,026	0,082
	p	0,357	<0,001	0,266	<0,001	0,562	0,066
Vit. C (mg/day)	r	0,129**	0,251***	0,116**	0,525***	0,079	,150***
	p	0,004	<0,001	0,010	<0,001	0,077	<0,001
Potassium (mg/day)	r	-0,035	0,130**	-0,019	0,362***	-0,026	0,077
	p	0,436	0,004	0,666	<0,001	0,568	0,084
Calcium (mg/day)	r	0,142**	0,461***	-0,007	0,274***	-0,018	-0,209***
	p	0,001	<0,001	0,882	<0,001	0,685	<0,001
Magnesium (mg/day)	r	0,043	0,415***	-0,032	0,402***	-0,007	0,117**
	p	0,333	<0,001	0,479	<0,001	0,869	0,009
Phosphorus (mg/day)	r	0,070	0,504***	-0,039	0,330***	-0,027	0,023
	p	0,116	<0,001	0,383	<0,001	0,543	0,603
Iron (mg/day)	r	-0,001	0,390***	-0,018	0,369***	-0,048	0,094*
	p	0,975	<0,001	0,688	<0,001	0,28	0,036
Zinc (mg/day)	r	0,055	0,379***	-0,025	0,300***	-0,067	-0,041
	p	0,218	<0,001	0,577	<0,001	0,132	0,356
Niacin (mg/day)	r	-0,029	0,238***	-0,034	0,345***	-0,069	0,119**
	p	0,518	<0,001	0,443	<0,001	0,124	0,008
PUFA (g/day)	r	-0,006	0,399***	-0,068	0,373***	-0,085	0,062
	p	0,892	<0,001	0,130	<0,001	0,057	0,164
MUFA (g/day)	r	0,122**	0,452***	-0,032	-0,003	-0,037	-0,142**
	p	0,006	<0,001	0,481	0,944	0,413	0,001

Values are correlation coefficients (r) with corresponding p-values; Spearman's and Pearson's correlation methods were used as appropriate. Statistical significance was indicated as *p<0.05, **p<0.01, and ***p<0.001. PUFA: polyunsaturated fatty acids; MUFA: monounsaturated fatty acids. α-TE: α-tocopherol equivalents.
